# Enhanced hexosamine metabolism drives metabolic and signaling networks involving hyaluronan production and *O*-GlcNAcylation to exacerbate breast cancer

**DOI:** 10.1038/s41419-019-2034-y

**Published:** 2019-10-23

**Authors:** Chatchadawalai Chokchaitaweesuk, Takashi Kobayashi, Tomomi Izumikawa, Naoki Itano

**Affiliations:** 1 Division of Life Sciences, Kyoto Sangyo University Graduate School, Kyoto, 603-8555 Japan; 2 Department of Molecular Biosciences, Faculty of Life Sciences, Kyoto Sangyo University, Kyoto, 603-8555 Japan; 3 Present Address: College of Pharmaceutical Sciences, Department of Pharmaceutical Sciences, Ritsumeikan University, Shiga, 525-8577 Japan

**Keywords:** Cancer metabolism, Cancer stem cells, Nutrient signalling, Glycobiology, Glycosylation

## Abstract

The hexosamine biosynthetic pathway (HBP) metabolically regulates dynamic cellular events by linking nutrient availability to numerous signaling networks. Significant alterations in the HBP are often associated with cancer pathogenesis. In this study, we investigated the molecular events underlying cancer pathogenesis associated with enhanced HBP flux. Multidimensional analysis of microarray datasets demonstrated up-regulation of genes encoding HBP enzymes in clinical breast cancers and revealed that co-expression of hyaluronan synthase 2 (HAS2) and glutamine:fructose-6-phosphate amidotransferase (GFAT), a rate-limiting enzyme of the HBP, was strongly correlated with a poor prognosis in advanced cancer patients. Consistently with the clinical data, comparative analyses of distinct breast cancer mouse models demonstrated enhancement of the HBP gene expression in primary carcinoma cells, with elevation of Has2 expression and hyaluronan production in aggressive breast cancer cells. The silencing of GFAT reduced CD44^high^/CD24^low^ cancer stem cell (CSC)-like subpopulations, aldehyde dehydrogenase-positive cell populations, and mammosphere size, which were further diminished by gene targeting of Has2. *Has2* gene disruption reduced the in vivo growth of aggressive cancer cells and attenuated pro-tumorigenic Akt/GSK3β/β-catenin signaling and cisplatin resistance. Overall protein *O*-GlcNAcylation was also elevated in association with HBP enhancement in aggressive cancer cells, and the modification exhibited overlapping but distinct roles from the hyaluronan signal in the regulation of CSC-like features. The current data therefore demonstrate that enhanced hexosamine metabolism drives pro-tumorigenic signaling pathways involving hyaluronan and *O*-GlcNAcylation in aggressive breast cancer.

## Introduction

Cancer cells reprogram metabolic pathways to optimally meet their energy and nutrient requirements. The most prominent metabolic alterations in cancer are an increase in glucose uptake and elevation of aerobic glycolysis, termed the Warburg effect^1,2^. With recent advances in metabolomics, the understanding of how metabolic reprogramming is linked to malignant transformation has expanded greatly^3,4^. Emerging evidence has also shown that metabolic shifts are an important factor in sustaining the self-renewing state of cancer stem cells (CSCs) responsible for tumor initiation, growth, and recurrence^5–7^. To date, however, there is limited knowledge on how the interplay between metabolic and signaling networks governs cancer development and progression.

The nutrient-sensing hexosamine biosynthetic pathway (HBP) is a glucose metabolic pathway branching off from main glycolysis^8^. The HBP synthesizes uridine diphosphate-*N*-acetylglucosamine (UDP-GlcNAc), which serves as a key metabolite essential for multiple protein glycosylations, glycosaminoglycan biosynthesis, and cellular signaling through protein *O*-GlcNAcylation. *O*-GlcNAcylation is a post-translational modification that transfers a single *O*-GlcNAc moiety from UDP-GlcNAc to serine/threonine residues of proteins^9^. *O*-GlcNAcylation is tightly regulated by the *O*-GlcNAc cycling enzymes *O*-GlcNAc transferase (OGT) and *O*-GlcNAcase (OGA). The modification occurs in a wide spectrum of intracellular proteins and regulates various distinct cellular processes, including transcription, translation, signal transduction, epigenetic regulation, and proteasomal degradation^9^. Given the diverse roles of *O*-GlcNAcylation, a potential link between hyper-*O*-GlcNAcylation and cancer progression has been proposed^10^.

Recent evidence has indicated a central role of the HBP in cancer metabolic rewiring and a close association of cancer development with enhanced HBP flux^11^. Elevated HBP enzyme expression has been detected in multiple human cancers. Itkonen et al. reported that UDP-GlcNAc pyrophosphorylase 1 (UAP1), the last enzyme in the pathway, was highly expressed in prostate cancer^12^. Oikari et al. also described the up-regulation of glutamine:fructose-6-phosphate amidotransferase (GFAT), a rate-limiting enzyme of the HBP, in human breast cancer biopsies^13^, whereby elevated GFAT expression was in parallel with an increase in UDP-GlcNAc content and was strongly correlated with tumor hyaluronan (HA) levels.

HA is a simple glycosaminoglycan in the extracellular matrix whose biosynthesis is regulated by three HA synthases (HAS1–3) that link UDP-GlcNAc and UDP-glucuronic acid (UDP-GlcUA) substrates^14^. There is considerable evidence correlating the degree of HA accumulation with a poor prognosis in advanced cancer patients^15–18^. Our animal studies have furthermore demonstrated that transgenic mice exhibiting HA overproduction in mammary tumors rapidly developed aggressive breast carcinomas, in which plastic cancer cells reverted to stem-cell states^19–22^.

HBP flux appears to influence cancer development and progression by controlling UDP-GlcNAc dynamics. However, the mechanisms underlying the cancer pathogenesis associated with enhanced HBP flux have not been fully elucidated. This study investigates the roles of the HBP and its downstream signals in breast cancer to uncover that enhanced HBP exacerbates cancer by driving metabolic and signaling networks involved in HA production and *O*-GlcNAcylation.

## Results

### Elevated expression of HBP genes in clinical breast cancers

Oncomine microarray gene expression datasets were initially analyzed across different types of clinical breast cancers for the expression of genes encoding HBP enzymes, including GFAT, glucosamine-phosphate *N*-acetyltransferase 1 (GNPNAT1), phosphoglucomutase 3 (PGM3), and UAP1, to investigate the molecular mechanisms underlying cancer pathogenesis associated with enhanced HBP flux^23,24^. The Ma Breast 4 dataset displayed significantly higher expression of all HBP genes in ductal breast carcinoma in situ epithelia (*n* = 9) than in normal samples (*n* = 14) (Supplementary Table S1). Databases were further searched for the expression of HBP enzymes across several datasets (Supplementary Table S1). Eight of 11 datasets showed that the expression of GFAT (GFAT1/2) was elevated over 1.5-fold in various types of breast cancers compared with normal samples. GFAT expression was often up-regulated together with GNPNAT1 and UAP1. In some datasets where GFAT expression was not significantly increased, one of GNPNAT1, PGM3, or UAP1 was highly expressed in cancer tissues. Thus, in silico gene expression analysis suggested the up-regulation of essential HBP enzymes in breast cancers across datasets.

### Co-expression of GFAT and HAS2 in aggressive breast cancers

**Fig. 1:**
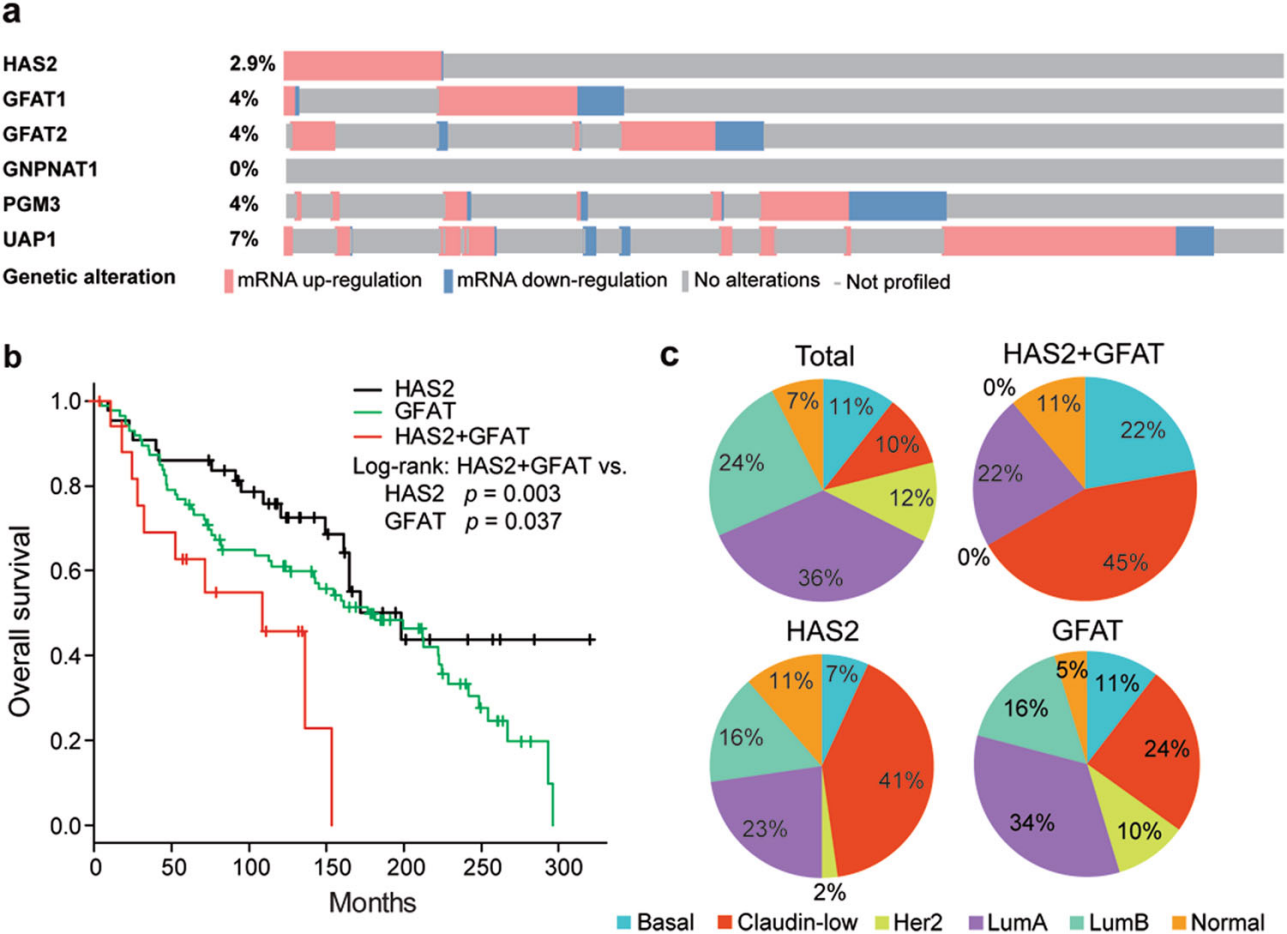
Co-expression of GFAT and HAS2 in breast cancer patients. **a** The mRNA expression data of HBP and *HAS2* genes were extracted from TCGA METABRIC datasets through cBioPortal and presented as OncoPrint for 2509 cases. Color coding indicates gene expression (red: up-regulation, blue: down-regulation). **b** Overall survival curves of breast cancer patients categorized according to the expression of HAS2 and GFAT (GFAT1 and 2). The HAS2^high^/GFAT^high^ group (HAS2 + GFAT: *n* = 18) was compared with the HAS2^high^ group (HAS2: *n* = 44) or GFAT^high^ group (GFAT: *n* = 86). **c** Distribution of the intrinsic molecular subtypes of breast cancer for HAS2 and GFAT mRNA expression. HAS2 positivity was enriched in the most aggressive subtypes, basal-like and claudin-low, and more prominent in combination with GFAT positivity (*n* = 1898 in total, 44 in HAS2, 86 in GFAT, and 18 in HAS2 + GFAT)

Since the significance of HA in tumor development has been highlighted by several pathological and experimental studies, we focused on this polysaccharide and its metabolism that is dynamically regulated by HBP flux. The genetic status of the three *HAS* genes was investigated in The Cancer Genome Atlas (TCGA) breast cancer database using cBioPortal (http://www.cbioportal.org/), which revealed that *HAS2* amplification was significantly higher in breast cancer across 5 datasets (Supplementary Fig. S1a). We next addressed the association between *HAS2* amplification and overall survival in breast cancer patients. Kaplan–Meier analysis of 5071 patients demonstrated that *HAS2* amplification was significantly correlated with shorter overall survival (Supplementary Fig. S1a). To further identify relationships between histological subtypes and genetic alterations, samples in TCGA PanCancer Atlas dataset comprising 1070 patient cases were evaluated^25^. *HAS2* was amplified in 13% of all breast cancers and 25% of metaplastic breast cancers, the latter being rare and aggressive variants (Supplementary Fig. S1b). In accordance with the gene amplification results, *HAS2* was transcriptionally active in aggressive metaplastic breast cancer (Supplementary Fig. S1c). Relationships between *HAS2* expression patterns and patient clinicopathological attributes were then examined using Molecular Taxonomy of Breast Cancer International Consortium (METABRIC) datasets (*n* = 2509, Fig. 1a)^26,27^. Gene expression profiling suggested a significant correlation (r = 0.4, *p* < 0.05) between GFAT (GFAT1/2) and HAS2 expression. Kaplan–Meier survival analysis of breast cancer patients (*n* = 148) demonstrated co-expression of GFAT and HAS2 to be more significantly associated with worse overall patient survival than the respective expression of GFAT or HAS2 alone (Fig. 1b). Multidimensional data analysis therefore indicated that co-expression of GFAT and HAS2 predicted a poor outcome.

Gene expression patterns were further assessed using METABRIC datasets of intrinsic molecular subtypes of breast cancer (luminal A, luminal B, normal breast-like, HER2-enriched, claudin-low, and basal-like). Of note, HAS2 positivity was enriched in basal-like and claudin-low subtypes, which became more prominent in combination with GFAT positivity (Fig. 1c). Considering that the basal-like and claudin-low subtypes belong to the group of triple-negative breast cancer having a high incidence of recurrence and metastasis, the co-expression of GFAT and HAS2 may confer aggressiveness in human breast cancer.

### Elevated expression of HBP genes, hyper *O*-GlcNAcylation, and increased HA production in a mouse model of aggressive breast cancer

**Fig. 2:**
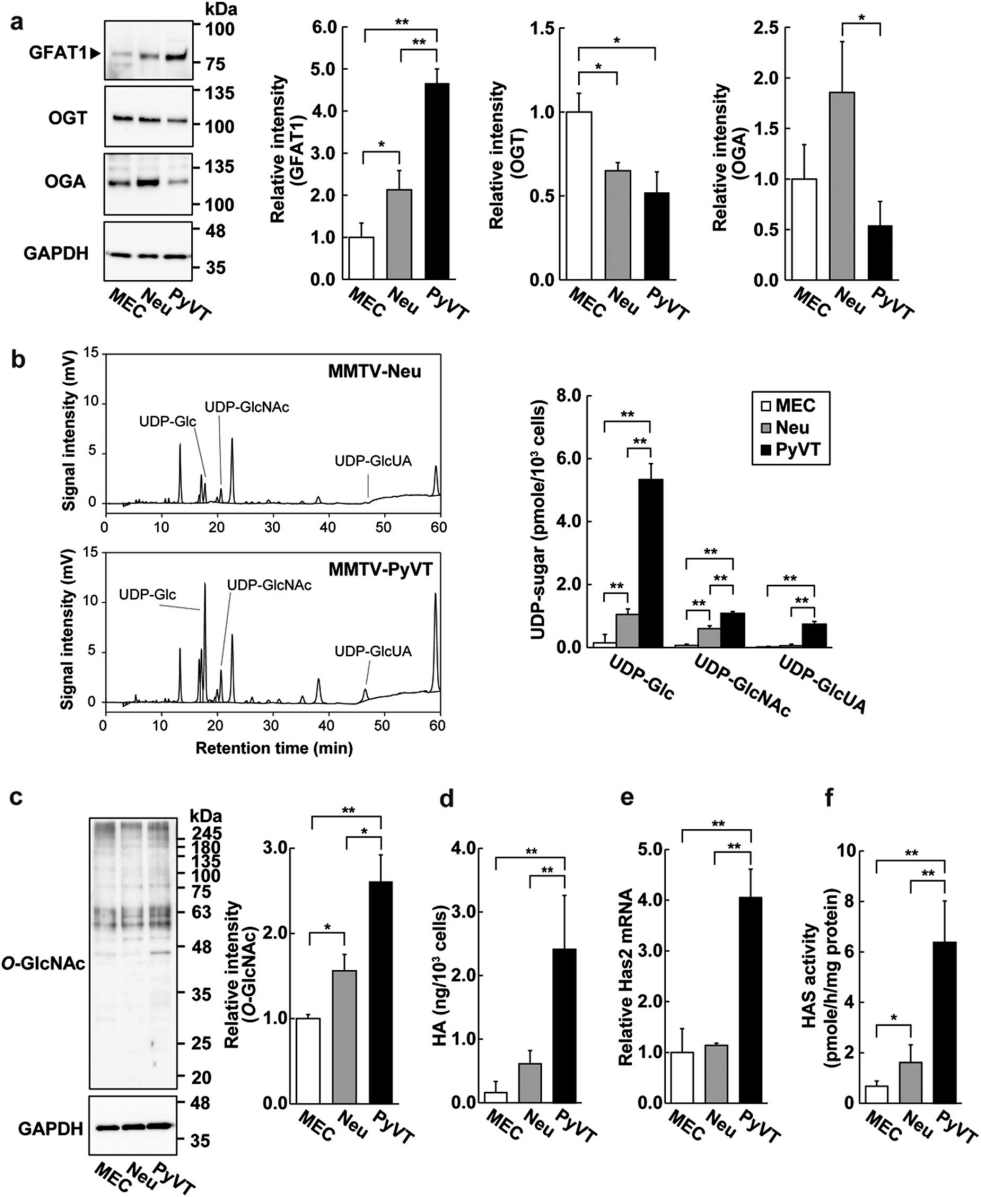
Protein *O*-GlcNAcylation and HA production in primary breast cancer cells. **a** Western blot analysis of GFAT1, OGT, and OGA. Cell lysates from MECs, MMTV-Neu (Neu), and MMTV-PyVT (PyVT) cells were subjected to western blot analysis. GAPDH was used as an internal control. Band intensities were quantified by densitometric analysis using ImageJ software and standardized with respect to GAPDH. Data represent the mean ± S.D. of three independent experiments. **b** HPLC analysis of UDP-sugars. Cellular levels of UDP-Glucose (UDP-Glc), UDP-GlcNAc, and UDP-GlcUA production in MECs, Neu and PyVT cells were monitored using HPLC. Data represent the mean ± S.D. of three independent experiments. **c** Western blot analysis of protein *O*-GlcNAcylation. Data represent the mean ± S.D. of three independent experiments. **d** HA contents in the conditioned medium of MECs, Neu, and PyVT cells were analyzed by a competitive ELISA-like assay. Data represent the mean ± S.D. of six independent experiments. **e** Relative expression of Has2 mRNA was analyzed by qRT-PCR. Data represent the mean ± S.D. of three independent experiments. **f** HAS activity was determined as described in the Materials and Methods. Data represent the mean ± S.D. of three independent experiments. *p < 0.05; **p < 0.01

We next sought to validate the findings from human clinical samples using murine breast cancer models. Mouse mammary tumour virus (MMTV)-Neu and MMTV-PyVT transgenic (Tg) mice were employed as murine breast cancer models for measuring the gene expression of HBP enzymes^28,29^. Both Tg lines develop palpable mammary tumors, while the MMTV-Neu phenotype differs from that of the MMTV-PyVT line recapitulating the progression of human mammary adenoma to late carcinoma stages and metastasizing primarily to the lymph nodes. Primary breast carcinoma cells were subjected to quantitative RT-PCR (qRT-PCR) analysis of HBP enzyme expression levels. The mRNA expression of GFAT1, GNPNAT1, PGM3, and UAP1 was significantly elevated in both primary carcinoma cell sets, particularly in the aggressive carcinoma cells derived from MMTV-PyVT tumors, as compared with the mammary epithelial cells (MECs) of control mice (Table 1), which was in fair agreement with the Oncomine database analysis. Significantly increased expression of GFAT1 mRNA was seen in MMTV-PyVT carcinoma cells, while GFAT2 mRNA expression in MMTV-Neu and MMTV-PyVT cells was markedly less than in controls. The increased expression of GFAT1 was validated at the protein level by western blot analysis (Fig. 2a), implying that the HBP was closely associated with breast cancer aggressiveness. To determine if the elevated expression of HBP enzymes increased the cellular pool of UDP-GlcNAc, UDP-sugars were monitored using high-performance liquid chromatography (HPLC). The cellular levels of UDP-GlcNAc as well as UDP-Glucose (UDP-Glc) and UDP-GlcUA were significantly increased in MMTV-PyVT carcinoma cells as compared with MEC and MMTV-Neu cells (Fig. 2b).

**Table 1:** mRNA expression of HBP enzymes in primary breast cancer cells

Gene	MEC	MMTV-Neu cell	MMTV-PyVT cell
GFAT1	1.00 ± 0.17	1.25 ± 0.19	1.67 ± 0.19^b^
GFAT2	1.00 ± 0.30	n.d.	0.02 ± 0.01^b^
GNPNAT1	1.00 ± 0.18	1.39 ± 0.14^a^	1.42 ± 0.21^a^
PGM3	1.00 ± 0.22	1.64 ± 0.22^a^	2.81 ± 0.33^b,c^
UAP1	1.00 ± 0.08	1.11 ± 0.04	1.86 ± 0.23^b,d^

Relative ± SD fold change of mRNA expression of three independent experiments

^a^*p*  < 0.05, ^b^*p*  < 0.01 compared with MEC. ^c^*p*  < 0.05, ^d^*p* < 0.01 compared with MMTV-Neu cell

*n.d*. not detected

The elevated expression of HBP enzymes and enhanced HBP flux may increase levels of protein *O*-GlcNAcylation and HA production by supplying additional UDP-GlcNAc. To ascertain this possibility, we examined the *O*-GlcNAc status of proteins by western blot analysis using anti-*O*-GlcNAc antibodies. Overall protein *O*-GlcNAcylation was significantly elevated in both malignant carcinoma cell lines as compared with MECs (Fig. 2c). It was noteworthy that the highest level of *O*-GlcNAcylation was detected in MMTV-PyVT cancer cells, which corroborated the increased level of UDP-GlcNAc. *O*-GlcNAc cycling enzyme expression was assessed by western blot analysis. In comparison with MECs, OGT expression was significantly down-regulated in both primary carcinoma cell lines (Fig. 2a). Given that cellular UDP-GlcNAc was significantly higher in these cancer cells than MECs (Fig. 2b), it is plausible that the levels of protein *O*-GlcNAcylation is predominantly controlled by HBP flux rather than OGT expression in these cells. In fact, a competitive GFAT antagonist, 6-diazo-5-oxo-l-norleucine (DON) decreased *O*-GlcNAc level in MMTV-PyVT cancer cells (Supplementary Fig. S2a), suggesting a crucial role of HBP flux in the determination of protein *O*-GlcNAcylation. The OGA expression was down-regulated in MMTV-PyVT cancer cells (Fig. 2a), which was inversely correlated with the highest level of *O*-GlcNAcylation. The low OGA expression in MMTV-PyVT cells was consistent with the Oncomine meta-analysis of breast cancer microarray database (Supplementary Table S2). On the other hand, the OGA expression in MMTV-Neu cancer cells was higher than that in MMTV-PyVT cells. The observations therefore imply that the OGA expression might be associated with the levels of protein *O*-GlcNAcylation.

When HA production was examined by a competitive ELISA-like assay, it was seen to be increased in MMTV-PyVT cancer cells but not in MMTV-Neu cells (Fig. 2d). Consistently with HA production level, *Has2* gene expression and HAS activity were elevated over 4-fold (*p* < 0.05) in MMTV-PyVT carcinoma cells (Fig. 2e, f), which strengthened the above notion that the coordinated up-regulation of both GFAT and HAS2 expression might confer aggressiveness in human breast cancer. Although gene expression profiles and HAS activity suggested that the up-regulation of Has2 was primarily responsible for the higher HA production in MMTV-PyVT cancer cells, enhanced HBP flux was also suspected to augment in the production of HA as HA level was decreased by DON exposure and rescued by d-Glucosamine (GlcN) (Supplementary Fig. S2b).

### HA production promotes CSC-like features and aggressive tumor growth

**Fig. 3:**
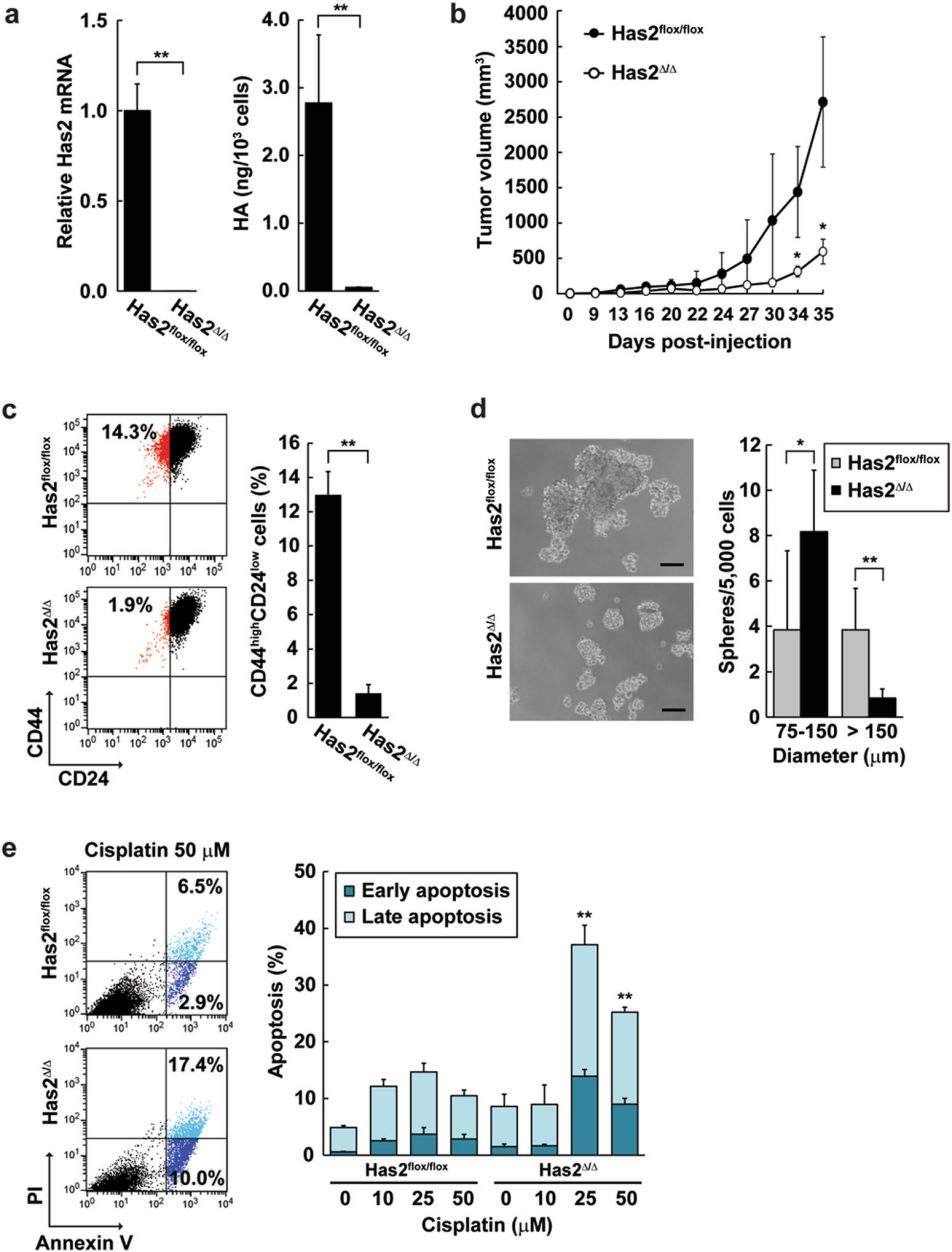
A deficiency in tumoral HA biosynthesis suppresses tumor growth and CSC-like phenotypes. **a** Has2 expression and HA production in Has2-deficient breast cancer cells. Total RNA samples isolated from Has2-deficient Has2^Δ/Δ^ and control Has2^flox/flox^ cells were subjected to qRT-PCR. The expression of Has2 was almost completely suppressed in Has2^Δ/Δ^ cells. HA content in the conditioned medium of Has2^Δ/Δ^ and Has2^flox/flox^ cells was measured by a competitive ELISA-like assay. HA content was markedly diminished in Has2-deficient Has2^Δ/Δ^ cells. Data represent the mean ± S.D. of three independent experiments. **p < 0.01. **b** Xenograft tumor growth of Has2-deficient breast cancer cells. Has2-deficient Has2^Δ/Δ^ and control Has2^flox/flox^ cells were inoculated at 1 × 10^6^ cells into the mammary fat pads of BALB/c nude mice. Tumor volume was measured every 2−4 days for 35 days. Data represent the mean ± S.D. (*n* = 6). *p *<* 0.05 versus control Has2^flox/flox^ cells. **c** Flow cytometric analysis of the CD44^high^/CD24^low^ subpopulation in Has2-deficient breast cancer cells. Has2-deficient Has2^Δ/Δ^ and control Has2^flox/flox^ cells were analyzed for CD24 and CD44 expression by flow cytometry. Data represent the mean ± S.D. of three independent experiments. **p < 0.01. **d** Mammosphere formation of Has2-deficient Has2^Δ/Δ^ and control Has2^flox/flox^ cells. Representative images of mammospheres were taken and mammosphere number was counted under a phase-contrast microscope. Scale bar: 100 µm. Data represent the mean ± S.D. of six independent experiments. *p < 0.05, **p < 0.01. **e** Cisplatin-induced apoptosis in Has2-deficient Has2^Δ/Δ^ and control Has2^flox/flox^ cells. Cells treated with 0-50 µM cisplatin for 16 h were stained with fluorescent Annexin V and PI, and then analyzed by flow cytometry. Early and late apoptotic cells were represented as Annexin V^+^/PI^-^ or Annexin V^+^/PI^+^ subpopulation, respectively. Data represent the mean ± S.D. of four independent experiments. **p < 0.01 Has2^Δ/Δ^ versus control Has2^flox/flox^ cells

To gain a better understanding of HA function in PyVT-induced carcinogenesis, MMTV-PyVT cancer cells harboring homozygous Has2 floxed alleles (Has2^flox/flox^ cells) were compared with Has2-deficient Has2^Δ/Δ^ cells. As evidenced by our previous study^30^, Cre-mediated recombination of the Has2 locus induced an almost complete failure of Has2 expression and HA production in Has2^Δ/Δ^ cells (Fig. 3a). When Has2-deficient Has2^Δ/Δ^ cancer cells were then transplanted into the mammary fat pads of nude mice, tumor growth was significantly suppressed as compared with that of the control Has2^flox/flox^ group (Fig. 3b). These data highlighted the significance of HA in cancer growth.

CSCs are believed to drive cancer growth and progression through aberrant self-renewal and the generation of heterogeneous cancer cell lineages^31^. CSC-like cells were assessed for the expression of CD44 and CD24 by flow cytometric analysis before and after deletion of the *Has2* gene. Has2-deficient Has2^Δ/Δ^ cancer cells exhibited a markedly reduced CD44^high^/CD24^low^ CSC-like subpopulation as compared with control Has2^flox/flox^ cancer cells (Fig. 3c). Aldehyde dehydrogenase-positive (ALDH^+^) populations from multiple types of cancers have been demonstrated to be enriched in cancer cells with stem-like characteristics and tumor-initiating ability^32,33^. In Aldefluor flow cytometry assays, Has2^Δ/Δ^ cancer cells displayed a smaller ALDH^+^ cell population than did control Has2^flox/flox^ cancer cells (Supplementary Fig. S3a). Breast CSCs have also been reported to form floating spherical colonies termed mammospheres to survive and proliferate in anchorage-independent conditions^34^. Control Has2^flox/flox^ cancer cells were capable of forming large mammospheres with high efficiency, whereas Has2-deficient Has2^Δ/Δ^ cancer cells mainly formed small mammospheres of 75–150 µm in diameter (Fig. 3d). CSCs often acquire resistance to anti-cancer drugs and are thereby thought to be responsible for tumor recurrence following treatment. Platinum-based chemotherapeutic drugs such as cisplatin are commonly used for treating metastatic triple-negative or basal-like breast cancers. Has2-deficient Has2^Δ/Δ^ and control Has2^flox/flox^ cancer cells were treated with cisplatin and the percentage of early and late apoptotic cells was determined by dual staining with fluorescent Annexin V and propidium iodide (PI). Early apoptotic cells showed Annexin V^+^/PI^−^ staining patterns, while late apoptotic cells exhibited Annexin V^+^/PI^+^ patterns. After exposure to cisplatin, a significant increase in early and late apoptotic cells was observed in Has2-deficient Has2^Δ/Δ^ cells (Fig. 3e). Taken together, these findings were in agreement with our previous study demonstrating a role of HA production in the regulation of CSC-like features and tumorigenesis.

### HA triggers the pro-tumorigenic Akt/GSK3β/β-catenin signaling pathway

**Fig. 4:**
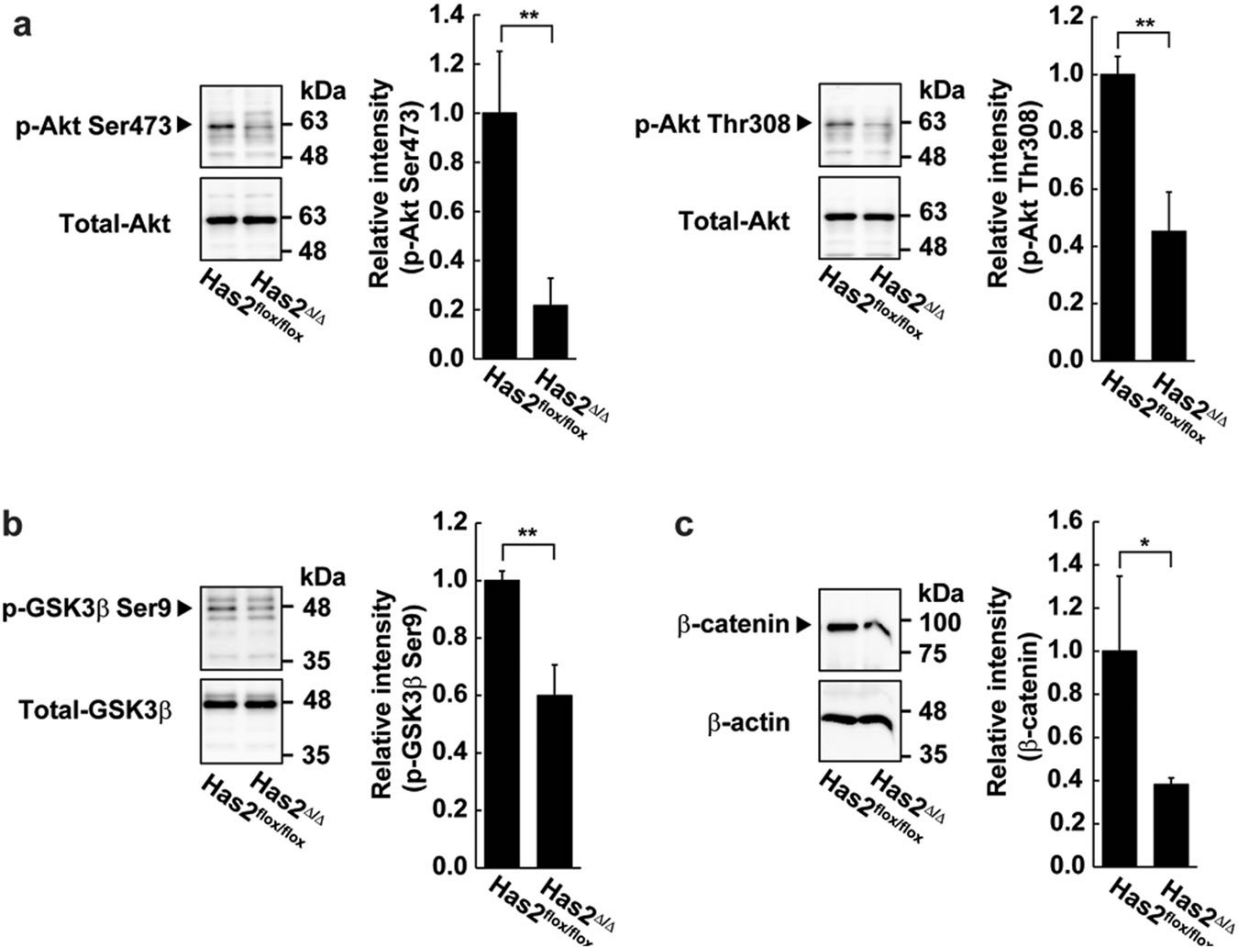
HA triggers the pro-tumorigenic signals. Cell lysates from Has2-deficient Has2^Δ/Δ^ and control Has2^flox/flox^ cells were subjected to western blot analysis to determine the phosphorylation of Akt (**a**) and GSK3β (**b**) and the expression of β-catenin (**c**). Total Akt, total GSK3β, or β-actin was used as an internal control. Band intensities were quantified by densitometric analysis using ImageJ software. Data represent the mean ± S.D. of three independent experiments. *p < 0.05; **p < 0.01

We next aimed to identify the signaling pathways involved in the pro-tumorigenic actions of HA. The phosphatidylinositol-3-kinase (PI3K)/Akt signaling pathway has emerged as a pro-tumorigenic signal, with recent studies showing links to CSC self-renewal^35,36^. Glycogen synthase kinase 3β (GSK3β) governs several signaling pathways associated with cancer progression and is inactivated upon phosphorylation in an Akt-dependent manner^37^. The Has2-deficient Has2^Δ/Δ^ cancer cells displayed greatly reduced Akt phosphorylation at both Ser473 and Thr308 as well as GSK3β phosphorylation at Ser9 as compared with control Has2^flox/flox^ cells (Fig. 4a, b). The phosphorylation of GSK3β inhibits its activity and prevents it from phosphorylating β-catenin, thus allowing the stabilization and nuclear translocation of β-catenin^38^. The stabilized β-catenin subsequently induces the epithelial-mesenchymal transition (EMT) crucial for the maintenance and expansion of CSCs. In accordance with the reduced phosphorylation of GSK3β, the expression of β-catenin was decreased in Has2-deficient Has2^Δ/Δ^ cancer cells (Fig. 4c).

### Coordinated actions of GFAT and Has2 on the regulation of CSC-like features

**Fig. 5:**
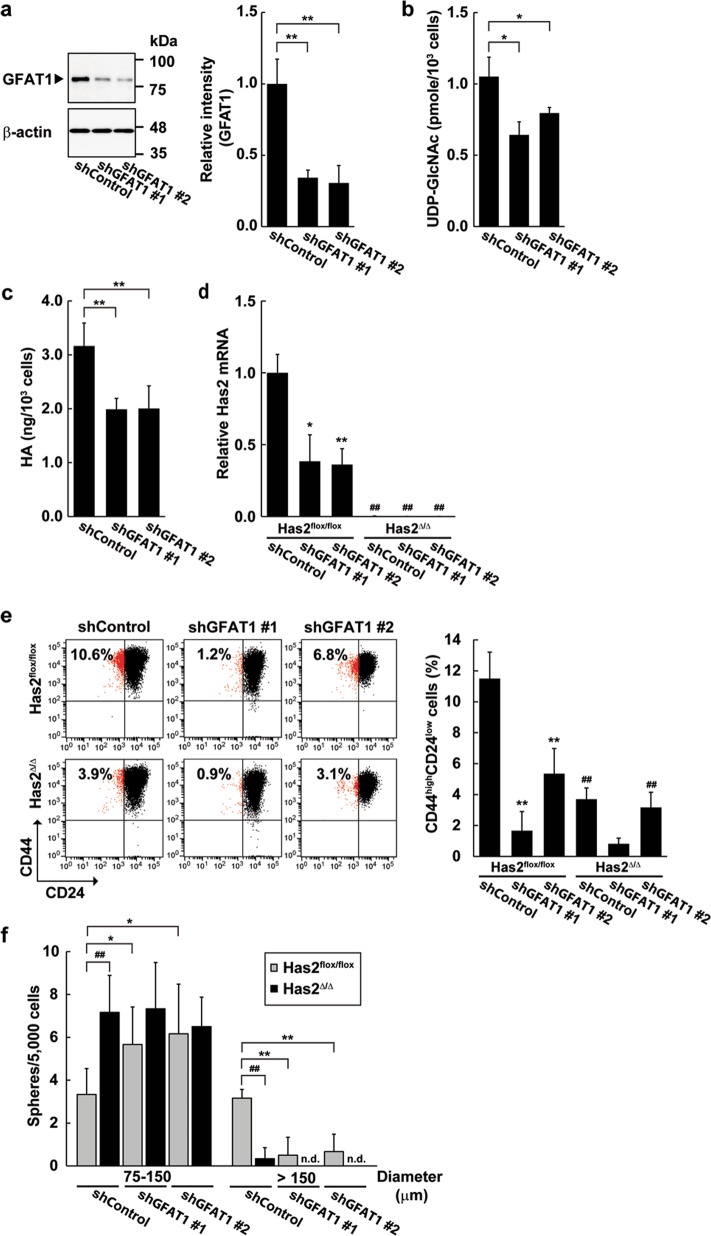
Coordinated action of GFAT and Has2 on the regulation of CSC-like features. **a** Western blot analysis of GFAT1 expression in GFAT1 knockdown cells. GFAT1 knockdown Has2^flox/flox^ cell pools were generated by transfection of lentivirus carrying GFAT1 shRNA and designated as shGFAT #1 and #2. Control cell pool was generated by a transfection of non-targeting shRNA and designated as shControl. Cell lysates were subjected to western blot analysis with anti-GFAT1 and anti-β-actin antibodies. Band intensities were quantified by densitometric analysis using ImageJ software and standardized with respect to β-actin. Data represent the mean ± S.D. of three independent experiments. **b** HPLC analysis of cellular UDP-GlcNAc levels in GFAT1 knockdown cells. Data represent the mean ± S.D. of three independent experiments. **c** HA production in GFAT1 knockdown cells was analyzed by a competitive ELISA-like assay. Data represent the mean ± S.D. of four independent experiments. **d** Relative expression of Has2 mRNA in GFAT1 knockdown/Has2-deficient cells. The expression in GFAT1 knockdown cells was analyzed before and after Has2 deletion by qRT-PCR. Data represent the mean ± S.D. of three independent experiments. **e** Flow cytometric analysis of the CD44^high^/CD24^low^ subpopulation in GFAT1 knockdown/Has2-deficient cells. CD24 and CD44 expression in GFAT1 knockdown cells was analyzed before and after Has2 deletion by flow cytometry. Data represent the mean ± S.D. of seven independent experiments. **f** Mammosphere formation of GFAT1 knockdown/Has2-deficient cells. Data represent the mean ± S.D. of six independent experiments. *p < 0.05; **p < 0.01 versus shControl. ^##^*p* < 0.01 versus corresponding control Has2^flox/flox^ cell. n.d. not detected

The multidimensional analysis of the clinical microarray datasets and comparative study of distinct breast cancer mouse models suggested GFAT and HAS2 co-expression in malignant breast cancer, which prompted us to examine whether the coordinated action of these enzymes was crucial for regulating CSC-like features. For this purpose, we silenced *GFAT1* gene expression in Has2^flox/flox^ cancer cells. A shRNA against murine GFAT1 mRNA was introduced into Has2^flox/flox^ cells by means of a lentiviral vector. The transduced cells were analyzed for endogenous GFAT1 protein level by western blot analysis (Fig. 5a). GFAT1 knockdown decreased its protein expression by ~50% as compared with Has2^flox/flox^ cells with control shRNA. Consistently with the decreased GFAT1 expression, cellular UDP-GlcNAc levels were lowered in the GFAT1 knockdown cells (Fig. 5b). Interestingly, GFAT1 knockdown significantly reduced *Has2* gene expression as well as HA production (Fig. 5c, d), suggesting GFAT-dependent regulation of Has2 expression. GFAT1 knockdown cells were analyzed for the expression of CD44 and CD24 and ALDH activity by flow cytometric analyses. GFAT1 silencing reduced both the CD44^high^/CD24^low^ CSC-like subpopulation and the ALDH^+^ cell population (Fig. 5e and Supplementary Fig. S3b). Similarly to *Has2* gene deletion, GFAT silencing reduced mammosphere size as the number of small mammospheres increased (Fig. 5f). GFAT1 knockdown cells were then analyzed before and after *Has2* gene disruption for the expression of CD44 and CD24 and mammosphere formation (Fig. 5d–f). Has2 deletion showed more remarkable effects on the attenuation of the CD44^high^/CD24^low^ CSC-like subpopulation than did GFAT1 knockdown alone (Fig. 5e). Compared with GFAT single knockdown, both the size and number of large mammospheres over 150 µm in diameter were reduced by the combination of GFAT knockdown and Has2 knockout (Fig. 5f). These results suggest the coordinated actions of GFAT1 and Has2 on the regulation of CSC-like phenotypes.

### HA and *O*-GlcNAcylation signaling pathways play overlapping but distinct roles in the regulation of CSC-like phenotypes

**Fig. 6:**
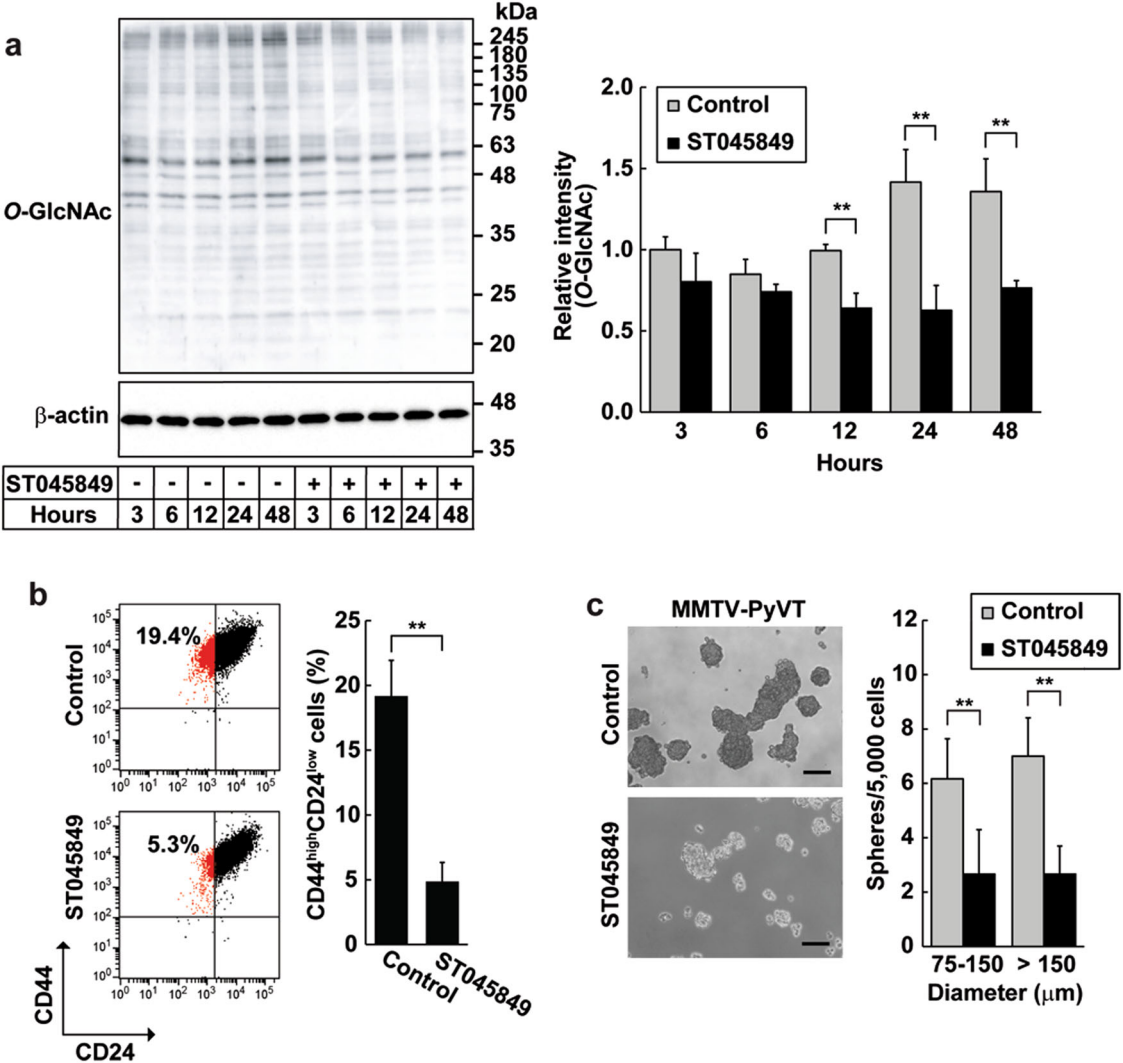
Significance of protein *O*-GlcNAcylation in the regulation of CSC-like features. **a** Western blot analysis for protein *O*-GlcNAcylation after inhibition of OGT. MMTV-PyVT cancer cells were treated with an OGT inhibitor (50 µM ST045849) for the indicated times (3–48 h). Western blot analysis was conducted to detect *O*-GlcNAcylated proteins. β-actin was used as an internal control. Band intensities were quantified by densitometric analysis using ImageJ software and standardized with respect to β-actin. Data represent the mean ± S.D. of three independent experiments. **p < 0.01 versus untreated control cells. **b** Flow cytometric analysis of the CD44^high^/CD24^low^ subpopulation after OGT inhibition. MMTV-PyVT cancer cells were treated with 50 µM ST045849 for 7 days and analyzed for CD24 and CD44 expression by flow cytometry. Data represent the mean ± S.D. of three independent experiments. **p < 0.01 versus untreated cells. **c** Mammosphere formation after OGT inhibition. MMTV-PyVT cancer cells were seeded into a 24-well low-attachment plate and treated with 50 µM ST045849 for 7 days. Representative images of mammospheres were taken and mammosphere number was counted under a phase-contrast microscope. Scale bar: 100 µm. Data represent the mean ± S.D. of three independent experiments. **p *<* 0.01 versus untreated cells

**Fig. 7:**
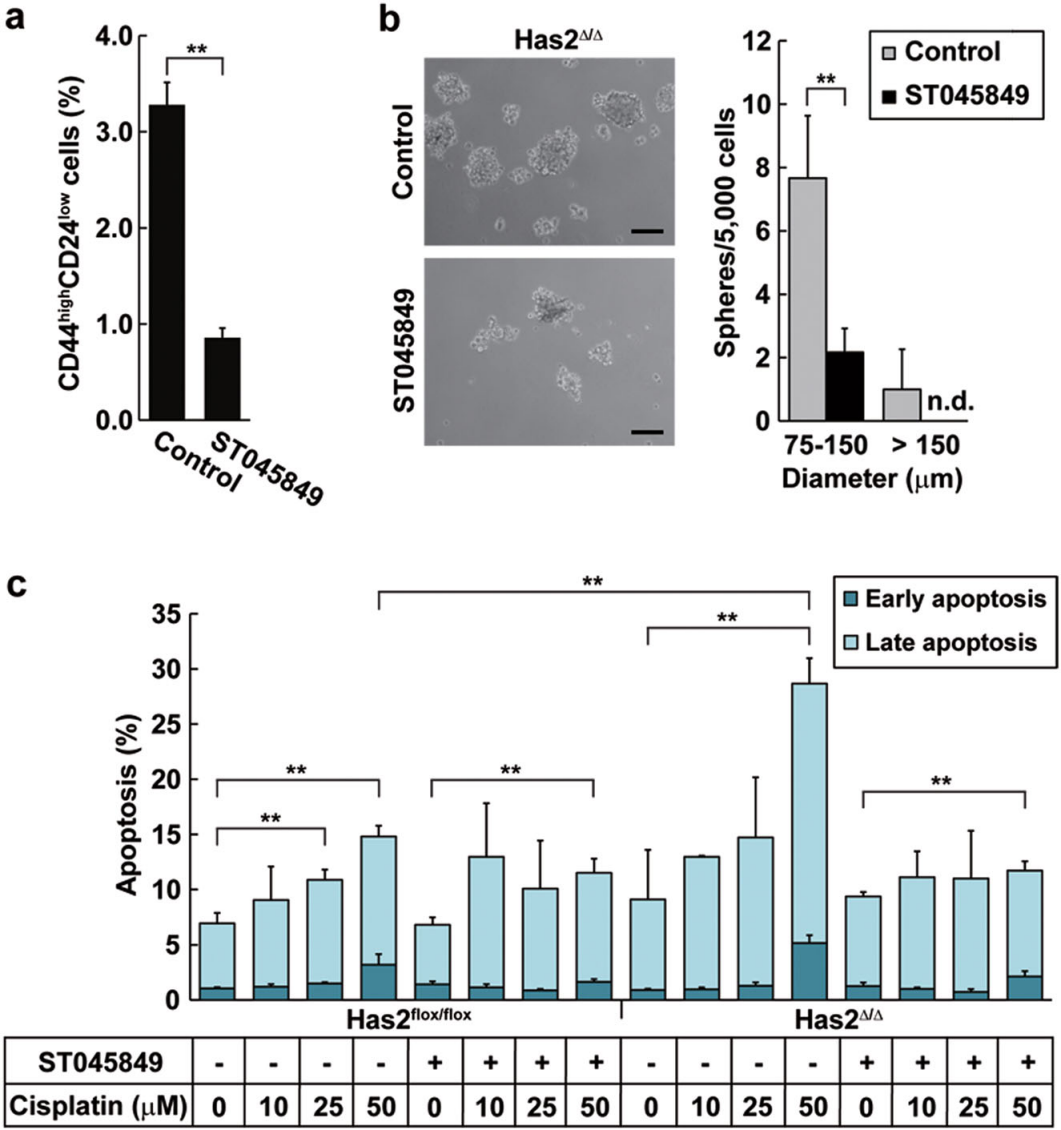
HA and *O*-GlcNAcylation signaling pathways play overlapping but distinct roles in the regulation of CSC-like phenotypes. **a** Flow cytometric analysis of the CD44^high^/CD24^low^ subpopulation after OGT inhibition. Has2-deficient Has2^Δ/Δ^ cells were treated with 50 µM ST045849 for 7 days and analyzed for CD24 and CD44 expression by flow cytometry. Data represent the mean ± S.D. of three independent experiments. **p < 0.01 versus untreated cells. **b** Mammosphere formation after OGT inhibition. Has2-deficient Has2^Δ/Δ^ cells were seeded into 24-well low-attachment plate and treated with 50 µM ST045849 for 7 days. Representative images of mammospheres were taken and mammosphere number was counted under a phase-contrast microscope. Scale bar: 100 µm. Data represent the mean ± S.D. of six independent experiments. n.d. not detected. **p < 0.01 versus untreated cells. **c** Cisplatin-induced apoptosis in Has2-deficient Has2^Δ/Δ^ and control Has2^flox/flox^ cells. Has2^Δ/Δ^ and Has2^flox/flox^ cells were treated with 50 µM ST045849 and cisplatin for 16 h. The treated cells were stained with fluorescent Annexin V and PI and then analyzed by flow cytometry. Early and late apoptotic cells were represented as Annexin V^+^/PI^-^ or Annexin V^+^/PI^+^ subpopulation, respectively. Data represent the mean ± S.D. of three independent experiments. **p < 0.01

The HBP serves as a key metabolic pathway essential for signaling networks involving protein *O*-GlcNAcylation. Hence, we investigated whether protein *O*-GlcNAcylation regulated CSC-like features as a HBP downstream signal. When the selective OGT inhibitor ST045849 was applied to MMTV-PyVT cancer cells ordinarily exhibiting hyper-*O*-GlcNAcylation, the overall levels of protein *O*-GlcNAcylation became significantly attenuated compared with the untreated control group (Fig. 6a). Consistently with the reduced *O*-GlcNAcylation, pharmacological inhibition greatly diminished the CD44^high^/CD24^low^ CSC-like subpopulation and the number of mammospheres (Fig. 6b, c). Moreover, OGT inhibition strengthened the suppressive effect of a Has2 deficiency (Fig. 7a, b). In contrast, the ALDH^+^ cell population was scarcely affected by OGT inhibitor treatment (Supplementary Fig. S3c). The above results therefore indicate that HBP flux comprehensively regulates CSC-like features by driving HA and *O*-GlcNAcylation signaling pathways.

A synergistic effect of cisplatin and an OGT inhibitor was then evaluated by Annexin V apoptosis assay. The rate of apoptotic cells was unchanged after exposure to cisplatin despite co-treatment with OGT inhibitor (Fig. 7c). To our surprise, the OGT inhibition almost completely rescued cisplatin resistance that had been suppressed by Has2 deficiency (Fig. 7c), implying that the hypo-*O*-GlcNAcylation may abolish the therapeutic efficacy of HA signal blockade against cisplatin resistance.

## Discussion

The HBP has emerged as a nutrient sensor that integrates nutrient availability with numerous cellular signaling pathways. The present study provided several lines of evidence that enhanced HBP flux drove metabolic and signaling networks involving HA and *O*-GlcNAcylation in aggressive breast cancer: (1) essential enzymes in the HBP were up-regulated in breast cancer cells, which was in agreement with findings in human clinical samples; (2) the *HAS2* gene was amplified in human breast cancers, and the expression of this gene was associated with aggressive types of breast cancer; (3) co-expression of HAS2 and GFAT was correlated with poor prognosis in advanced breast cancer patients and cancer cell aggressiveness; (4) GFAT suppression diminished CSC-like features along with the reduction of HA production and protein *O*-GlcNAcylation; (5) gene targeting of Has2 significantly suppressed CSC-like phenotypes and xenograft tumor growth and attenuated the pro-tumorigenic Akt/GSK3β/β-catenin signal and anti-cancer drug resistance; (6) HA and *O*-GlcNAcylation signaling pathways play overlapping but distinct roles in the regulation of CSC-like phenotypes.

HA has been shown to provide a favorable microenvironment for the self-renewal and maintenance of CSCs. Bourguignon et al. demonstrated that interactions between HA and its receptor, CD44v3, propagated cancer stemness of human head and neck squamous cell carcinoma cells via the stem-cell factors Oct4, Sox2, and Nanog^39^. Ohno et al. recently reported that HA-CD44 interactions regulated the spheroid formation and maintenance of cancer-initiating cells in malignant mesothelioma^40^. HA in the tumor microenvironment also indirectly affected CSC self-renewal by influencing the behavior of stromal cells^41^. In contrast, we have recently found that HA production may metabolically regulate CSC-like features^30^, although the precise mechanism remains unknown. Since HA is normally synthesized at the plasma membrane using donor substrates, its overproduction can directly reduce cytosolic UDP-GlcUA and UDP-GlcNAc levels. Therefore, HA-overproducing cells may accelerate HBP flux to balance synthesis and consumption of UDP-GlcNAc. This was evident from the fact that forced expression of Has2 in MMTV-Neu cancer cells significantly accelerated HBP flux^30^. The HBP senses the reduction of cytosolic UDP-GlcNAc availability and accelerates the rate-limiting step catalyzed by GFAT, thus serving to provide sufficient UDP-GlcNAc^8^. Therefore, the positive feedback loop between HBP changes and HA production may act in a system amplifying a series of signals triggered by accelerated HBP flux. It will be of interest to further explore whether HA regulates CSC-like features not only by evoking signals in a receptor-mediated fashion, but also by modulating cellular signaling via its biosynthesis.

The present study indicated that co-expression of HAS2 and GFAT was highly associated with the aggressive cancer subtypes and strongly correlated with a poor prognosis in advanced cancer patients. In agreement with the clinical data, GFAT1 and Has2 co-expression was evident in aggressive MMTV-PyVT cancer cells. The coordinated regulation of *GFAT* and *Has2* gene expression was further supported by the result that GFAT1 knockdown in MMTV-PyVT mammary carcinoma cells significantly reduced *Has2* gene expression. The promoter region of the *HAS2* gene contains functional response elements for several transcription factors, including CREB, STAT3, FOXO1, SP1, and YY1. Dynamic HBP flux regulates the transcriptional activation of these factors through *O*-GlcNAcylation. Jokela et al. have demonstrated that GFAT1 silencing in HaCaT keratinocytes increased HAS2 expression by limiting the *O*-GlcNAc-modification of SP1 and YY1^42^. The difference in the cellular responsiveness of mammary carcinoma cells and keratinocytes to GFAT1 silencing may be attributed to the expression patterns of transcriptional regulators in these cells. HAS2-AS1, a natural antisense transcript, has also been reported to regulate HAS2 transcription by altering the chromatin structure around the HAS2 proximal promoter^43^. *O*-GlcNAcylation modulates HAS2-AS1 promoter activation by recruiting the NF-κB subunit p65. Therefore, both HBP flux and downstream *O*-GlcNAcylation may multiply regulate HAS2 expression by context-dependent mechanisms via transcription factors and HAS2-AS1.

The suppressive effects of GFAT1 silencing on mammosphere formation resembled those of a Has2 deficiency, which further strengthened the conclusion that GFAT and Has2 coordinately regulate CSC-like features. GFAT1 silencing was sufficient to reduce HA production, but only had a moderate effect on suppressing *O*-GlcNAcylation (Fig. 5c and Supplementary Fig. S4a). Given these results, HA signaling may be dominantly affected by changes in HBP flux while cellular *O*-GlcNAc levels are somewhat maintained within an optimal range. The existence of a feedback loop that maintains *O*-GlcNAc homeostasis has been demonstrated by the fact that the reduction of cellular UDP-GlcNAc level up-regulated OGT expression and down-regulated OGA expression^44,45^. However, this was not the case in our cancer cell system, because the expression levels of OGT and OGA were unaffected by GFAT1 silencing (Supplementary Fig. S4b). *O*-GlcNAc homeostasis is determined by the availability of donor and acceptor substrates as well as by OGT/OGA expression and activity. Since it is possible that HA biosynthesis and *O*-GlcNAcylation compete for the availability of a donor UDP-GlcNAc substrate, the different kinetics between the enzymes involved in both reactions may be critical for determining which signaling pathway is dominant to control mammosphere formation as a HBP downstream signal. The lowest *Km* of the OGT active subunit is almost five-fold less than that of Has2, supporting the notion that changes in HBP flux preferentially affect HA signaling over *O*-GlcNAcylation^46,47^.

Mammosphere number and size reflect the self-renewal and proliferation of mammary stem/progenitor cells, respectively^48,49^. We observed that while OGT inhibition markedly reduced the size and number of mammospheres, GFAT knockdown or *Has2* gene deletion only reduced their size. OGT inhibition further reduced the number of mammospheres whose size was diminished by a Has2 deficiency. Therefore, we postulate that *O*-GlcNAcylation modulates the key signals necessary for stem-cell self-renewal, whereas HA signaling promotes the proliferation of cells with sphere-forming ability. Collectively, enhanced HA and *O*-GlcNAcylation signals may complementarily or synergistically promote CSC-like features as downstream signals of the HBP. Although the mechanism of mammosphere size regulation is currently unclear, the PI3K/Akt survival signal triggered by HA may contribute to the proliferation and viability of stem-like cells in anchorage-independent conditions^50^.

CD44^high^/CD24^low^ and ALDH^high^ have been widely-accepted as CSC-like phenotypes in breast cancer. To date, however, the relationship between different phenotypes has not been clearly established. In the current study, OGT inhibition altered CD44/CD24 expression, but not ALDH activity; hence, these phenotypes may be regulated in an independent manner. Considering that a Has2 deficiency attenuated ALDH activity as well as the CD44^high^/CD24^low^ subpopulation, enhanced HA and *O*-GlcNAcylation signals may additively or synergistically promote the CD44^high^/CD24^low^ CSC-like phenotype.

A Has2 deficiency significantly suppressed the cisplatin resistance that was unaffected by OGT inhibition, suggesting distinct roles between HA and *O*-GlcNAcylation signaling pathways in cisplatin resistance. PI3K/Akt/mTOR pathway activation has been implicated in the cisplatin resistance of triple-negative breast cancer cells^51^. Considering that a Has2 deficiency attenuated both PI3K/Akt signaling and cisplatin resistance, the HA/PI3K/Akt signaling axis appears to be important for the acquisition of drug resistance. On the other hand, the hypo-*O*-GlcNAcylation induced by OGT inhibition almost completely rescued the cisplatin resistance that had been suppressed by a Has2 deficiency, suggesting that potent inhibition of *O*-GlcNAcylation may abolish the therapeutic efficacy of a HA signal blockade against cisplatin resistance. The contradictory action of *O*-GlcNAcylation on cisplatin resistance may therefore be attributed to aberrant hypo-*O*-GlcNAcylation arising from OGT inhibitor treatment. Hence, the development of CSC therapeutics based on *O*-GlcNAcylation inhibition requires careful consideration.

In conclusion, we uncovered that an enhanced HBP drove pro-tumorigenic signaling pathways involving HA and *O*-GlcNAcylation in aggressive breast cancer. Furthermore, the HA and *O*-GlcNAcylation signaling pathways exhibited overlapping but distinct roles in the regulation of CSC-like phenotypes. Designing the most effective and appropriate strategy towards the prevention and interception of such pro-tumorigenic signals may therefore contribute to the achievement of breast cancer elimination.

## Materials and methods

### Oncomine microarray database analysis

The expression patterns of HBP genes, including *GFAT1*, *GFAT2*, *GNPNAT1*, *PGM3*, and *UAP1*, were analyzed using the breast cancer datasets in the Oncomine Cancer Microarray database (https://www.oncomine.org). Briefly, the target genes were queried in the database and the results were filtered by selecting “breast cancer” and “cancer vs. normal”. Statistical comparisons were conducted using Oncomine algorithms. Details of the standardized normalization technique and statistical calculations are available on the Oncomine website.

### The cBioPortal for TCGA analysis

To evaluate the genetic status of the *HAS2* gene, breast cancer datasets from METABRIC (2509 samples)^27^, Mutational profiles of metastatic breast cancer (France, 2016; 216 samples)^52^, Metastatic Breast Cancer Project (Provisional, April 2018; 157 samples) (http://tcga-data.nci.nih.gov/tcga/), Breast Invasive Carcinoma (TCGA, PanCancer Atlas; 1084 samples)^53–58^, and Breast Invasive Carcinoma (TCGA, Provisional; 1105 samples) (https://www.mbcproject.org) were downloaded through the cBioportal web-based utility (http://www.cbioportal.org). The cBioPortal was used to explore the genetic alterations and gene expressions across the datasets^59^. Raw data of GFAT and HAS2 mRNA expression and clinical information from the METABRIC dataset (EGAS00001001753 from the European Genome-phenome Archive) were employed to evaluate the co-expression of these genes and mRNA expression Z-score associations with clinicopathological characteristics. The correlation between the gene expression of GFAT and/or HAS2 and the overall survival of breast cancer patients was also analyzed by EZR on R commander^60^ using the METABRIC array datasets with 148 cancer samples overexpressing either or both genes. The log-rank *p*-value and hazard ratio with 95% confidence interval were calculated as well.

### Primary breast carcinoma cells and cell culture conditions

All primary breast carcinoma cells used in this study were established as described previously^22,30^. Briefly, Has2^flox/flox^ mice were generated and backcrossed to FVB/N-Tg(MMTV-PyVT)634Mul/J mice. Has2^flox/flox^ breast carcinoma cells were established from primary mammary tumors that had developed spontaneously in Has2^flox/flox^ tumor model mice. Has2^flox/flox^ breast carcinoma cells were infected with the AxCANCre adenovirus carrying the Cre recombinase gene driven by a CAG promoter to generate Has2-deficient Has2^Δ/Δ^ cells. Has2^flox/flox^ cells infected with the AxCANLacZ adenovirus carrying the β-galactosidase (LacZ) gene served as a control (Has2^flox/flox^ cells). All cancer cells were grown in Dulbecco’s modified Eagle’s medium (DMEM) containing 10% fetal bovine serum (FBS) under the standard culture conditions of a humidified atmosphere of 95% air and 5% CO_2_ at 37 °C. Normal mouse MECs were isolated as described by Prater et al. with some modifications^61^. For the detection of phospho-Akt and GSK3β, Has2^flox/flox^ and Has2^Δ/Δ^ cancer cells were grown for 48 h in cell culture medium supplemented with 10% dialyzed FBS and 5.5 mM d-glucose.

### QRT-PCR

Total RNA from breast carcinoma cells was isolated using the Qiagen RNeasy mini kit (Qiagen, Germantown, MD). Complementary DNA was synthesized with the PrimeScript RT Reagent kit (Takara Bio, Shiga, Japan) according to the manufacturer’s instructions. For murine Has2 and GAPDH, qRT-PCR was performed as described previously^19^. The TaqMan gene expression assays (Applied Biosystems, Foster City, CA, USA) used were as follows: Assay ID: Mm01183874_m1 (GFAT1), Mm00496565_m1 (GFAT2), Mm00834602_mH (GNPNAT1), Mm01144498_m1 (PGM3), Mm01281909_m1 (UAP1), Mm00507317_m1 (OGT), and Mm00452409_m1 (OGA). The RT-PCR conditions for gene expression were as follows: one cycle at 94 °C for 30 s and 40 cycles at 94 °C for 3 s and 60 °C for 25 s. Relative mRNA expression was analysed by the comparative Ct method and normalized using GAPDH expression.

### Tumorigenicity assay

The control Has2^flox/flox^ and Has2-deficient Has2^Δ/Δ^ cells (1 × 10^6^ cells/injection) were suspended in Hanks’ balanced salt solution and injected into the mammary fat pads of BALB/c nude mice (*n* = 6 per group, 8-week-old female, CLEA Japan, Inc., Tokyo, Japan). Tumor size was recorded for 35 days after inoculation. Tumor diameter was measured every 2 to 4 days with digital calipers, and tumor volume was calculated by the formula: volume = (width)^2^ × length/2. Animal care and all experimental procedures using animal models were performed in biosafety level 2 animal facilities according to the established guidelines approved by the Kyoto Sangyo University ethics committee.

### Gene silencing with shRNA

Recombinant lentivirus particles were produced by Lenti-vpak lentivirus packaging kit (OriGene, Rockville, MD) with Lenti-X 293 T cell (Takara Bio, Kusatsu, Shiga, Japan) according to the manufacturer’s instruction. Lentivirus carrying the shRNA of murine GFAT1 (Gene ID 14583, OriGene) and control non-targeting shRNA were infected to Has2^flox/flox^ cells as described previously^30^. Transduced cells were then selected in the presence of 10 μg/ml puromycin and 50 μM d-Glucosamine for 14 days. To delete *Has2* gene, shGFAT and shControl cells were infected with AxCANCre and AxCANLacZ adenovirus, respectively, as described previously^30^.

### Apoptosis assay

One hundred thousand of Has2^flox/flox^ and Has2^Δ/Δ^ cells were cultured in 35 mm dish for 24 h and then treated with 0–50 μM cisplatin (Wako Purechemical Industries, Osaka, Japan) in DMEM containing 10% FBS for 16 h. After the treatment, the cells were stained with MEBCYTO Apoptosis Kit (MBL Co., Ltd., Nagoya, Japan) according to the manufacturer’s instruction and analysed by FACSCalibur (BD Biosciences, Franklin Lakes, NJ).

### Statistical analysis

The two-tailed Student’s *t*-test or Tukey’s multiple comparison test was used to determine the differences among means. All experiments were performed at least three times. Data were expressed as the mean ± standard deviation (S.D.) A *p*-value of less than 0.05 was considered statistically significant.

## Supplementary information

Supplementary information

Supplementary Table S1

Supplementary Table S2

Supplementary Figure S1

Supplementary Figure S2

Supplementary Figure S3

Supplementary Figure S4

Detailed Attribution of Authorship

Additional Detailed Attribution of Authorship

Reproducibility checklist
