# Supplementary material for: Enhanced hexosamine metabolism drives metabolic and signaling networks involving hyaluronan production and O-GlcNAcylation to exacerbate breast cancer
Source: Cell Death Dis. 2019 Oct 23;10(11):803. doi: 10.1038/s41419-019-2034-y (PMC6811536; doi:10.1038/s41419-019-2034-y)
Supplement: Supplementary file 1 — Supplementary information [file 41419_2019_2034_MOESM1_ESM.docx]

**Supplementary information**

**Supplementary materials and methods**

**Chemicals and reagents**

The following cell culture reagents were used: Dulbecco’s modified Eagle’s medium (DMEM) (Nacalai Tesque, Osaka, Japan), fetal bovine serum (FBS) (Biosera, Nuaille, France), penicillin-streptomycin (Wako Pure Chemical Industries, Osaka, Japan), glucose-free DMEM (Sigma-Aldrich, St. Louis, MO), serum-free DMEM/Ham’s F12 (Nacalai Tesque), bFGF (Wako Pure Chemical Industries, Osaka, Japan), EGF (Miltenyi Biotec, Bergisch Gladbach, Germany), and B27 (Gibco Life Technologies, Grand Island, NY, USA). DON was purchased from Sigma-Aldrich and ST045849 was from TimTec LLC (Newark, DE). The following antibodies were used: anti-*O*-GlcNAc (CTD110.6), anti-OGT (D1D8Q), anti-phospho-Akt (Thr308) (C31E5E), anti-phospho-Akt (Ser473) (D9E), anti-Akt, anti-phospho-Glycogen synthase kinase (GSK) 3β (Ser9), anti-GSK3β (27C10), anti-β-catenin (D10A8), anti-glyceraldehyde-3-phosphate dehydrogenase (GAPDH) (14C10), horseradish peroxidase (HRP)-conjugated anti-mouse IgG, and HRP-conjugated anti-rabbit IgG were purchased from Cell Signaling Technology (Danvers, MA). Anti-β-actin was purchased from Wako Pure Chemical Industries. Anti-GFAT1 and anti-OGA were purchased from Proteintech (Rosemont. IL). The phycoerythrin (PE)-conjugated anti-CD44 and FITC-conjugated anti-CD24 antibodies, biotin-conjugated anti-CD31 antibody (RA3-6B2), and biotin-conjugated anti-Ter119 antibody (TER-119) were purchased from eBioscience (San Diego, CA). The PE/Cy7-conjugated anti-EpCAM antibody was purchased from Biolegend (San Diego, CA). Streptavidin-Alexa 488 was purchased from ThermoFisher Scientific (Waltham, MA).

**Preparation of primary mouse mammary epithelial cells**

Normal mouse mammary epithelial cells were isolated as described by Prater *et al.* with some modifications^1^. Mammary glands (No. 3, 4, and 5 gland pairs) were dissected from 13–16-week-old FVB/NJcl mice (CLEA Japan Inc. Tokyo, Japan). The finely minced glands were digested in complete medium (DMEM/F12 containing 5% FBS, 5 μg/ml insulin, 5 ng/ml EGF, 1 μg/ml hydrocortisone, 50 μg/ml gentamycin, 100 U/ml penicillin, and 100 μg/ml streptomycin) containing 1 mg/ml collagenase (Wako Pure Chemical Industries) and 100 U/ml hyaluronidase (Nacalai Tesque) for 16 h at 37 °C. The mammary organoids were digested with 0.25% trypsin (Wako Pure Chemical Industries) and 1 mM EDTA in Hanks’s balanced salt solution (HBSS) (Wako Pure Chemical Industries), 5 U/ml dispase (Wako Pure Chemical Industries) in HBSS, and 1 mg/ml DNaseI (StemCell Technologies, Vancouver, Canada) followed by the lysis of red blood cells in NH_4_Cl. The single cell suspensions were collected with a 40 μm cell strainer (Corning, Steuben County, NY) and incubated in complete medium for 1 h at 37 °C. After blocking with 10% normal rat serum (Wako Pure Chemical Industries) in DMEM/F12, the mammary cells were stained with anti-CD31-biotin antibody (1:250 dilution) and anti-Ter119-biotin antibody (1:250) in phosphate buffer saline (PBS) containing 3% bovine serum albumin (BSA) for 1 h at 4 °C. The cells were then stained with streptavidin-Alexa 488 (1:200) and anti-EpCAM-PE/Cy7 (1:500) for 1 h at 4 °C. Cells were sorted using FACSMelody (BD Biosciences, Franklin Lakes, NJ) following filtration through a 42 μm nylon mesh. The high EpCAM-, low CD31-, low TER-119-expressing population was sorted as primary MECs. Total RNA or protein was immediately isolated from sorted MECs using an RNeasy mini kit (Qiagen, Germantown, MD) or RIPA buffer (Nacalai Tesque), respectively. For quantification of HA synthesis, a fraction of the sorted cells was cultured in 96-well plates coated with Matrigel (Wako Pure Chemical Industries).

**HA measurement and HA synthase assay**

HA concentrations were determined by a competitive ELISA-like assay, as described previously^2^. HAS activity was monitored in the cell-free HA synthesis system using UDP-[^14^C] GlcUA and UDP-GlcNAc as donors and a membrane-rich fraction of the cells as an enzyme source as described previously^3,4^.

**UDP-sugar quantification by HPLC**

UDP-sugar was quantified by Ion-pair reverse-phase HPLC methods as described by Nakajima *et al*. with some modifications^5^. Cellular extracts were prepared from 300,000 to 500,000 of each cell. The sample was subjected to ion-pair solid phase extraction using an Envi-Carb column (Supelco Inc, Bellefonte, PA). A Prominence HPLC system (Shimazu, Kyoto, Japan) was used for UDP-sugar quantification by an absorbance at 254 nm. Separation of UDP-sugars was performed at 40 °C on Inertsil ODS-3 column (250 × 4.6 mm internal diameter, 3 μm particle size; GL Science, Tokyo, Japan).

**SDS-PAGE and Western blotting**

SDS-PAGE and western blotting were performed as described previously^6^. MMTV-PyVT cancer cells were seeded at 2.5 × 10^5^ cells/well and cultured in 10% FBS/DMEM with or without 50 µM ST045849 for 3–48 h. Cells were harvested at indicated times, washed with cold PBS, and lysed in RIPA buffer (50 mM Tris-HCl, pH 7.6, 150 mM NaCl, 1% Nonidet P40, 0.5% sodium deoxycholate, 1 × protease inhibitor cocktail [EDTA free], and 0.1% SDS) (Nacalai Tesque). For detection of phosphorylated proteins, cells were lysed in RIPA buffer containing phosphatase inhibitor cocktail (Nacalai Tesque). Protein concentrations were determined by the Pierce Microplate BCA protein assay kit (Thermo Scientific) according to the manufacturer’s instructions. Equal amounts of whole cell lysates were subjected to SDS-PAGE on 8-12% polyacrylamide gels. After protein transfer to PVDF membranes (Millipore, Burlington, MA), the membranes were blocked with 0.1% Tween-TBST (50 mM Tris-HCl, pH 7.4, 138 mM NaCl, 2.7 mM KCl, and 0.1% Tween 20) containing 5% BSA (Nacalai Tesque) or 5% skim milk for 60 min at room temperature. The membranes were then probed with the primary antibody anti-*O*-GlcNAc (1:1000 dilution), anti-GFAT1 (1:1000), anti-OGT (1:1000), anti-OGA (1:3000), anti-p-Akt Ser473 (1:2000), anti-p-Akt Thr 308 (1:1000), anti-p-GSK3β Ser9 (1:1000), anti-β-catenin (1:2000), anti-Akt (1:1000), or anti-GSK3β (1:1000) at 4 °C overnight. Anti-β-actin antibody or anti-GAPDH antibody was used as an internal control. Excess antibodies were removed by washing in 0.1% Tween-TBST, with subsequent incubation with HRP-conjugated secondary antibodies (1:2000) for 60 min at room temperature. Signals were visualized using western blotting detection reagents (Wako Pure Chemical Industries). Chemiluminescent signals were observed on an ImageQuant LAS4000 Mini Luminescent image analyzer (GE Healthcare, Chicago, IL). Band intensities were quantified by densitometric analysis using ImageJ software (National Institutes of Health, Bethesda, MD).

**Flow cytometric analysis**

Flow cytometric analysis was performed as described previously^6^. Breast carcinoma cells were seeded at 1 × 10^5^ cells/35 mm dish and cultured at 37 °C for 24 h in DMEM containing 10% FBS. MMTV-PyVT cancer cells were treated with or without 50 µM ST045849 every 3 days and incubated for 7 days. Then, 5 × 10^5^ cells were harvested and washed in cold PBS supplemented with 1% FBS. Cell suspensions were incubated with 50 µl of 1% FBS-PBS containing PE-conjugated anti-CD44 (1:160 dilution) and FITC-conjugated anti-CD24 (1:50) antibodies for 60 min on ice in the dark. The cells were washed with 1% FBS-PBS by centrifugation, resuspened in cold 1% FBS-PBS, and then filtered through cell strainers. A total of 10,000 viable cells were analyzed using the FACSMelody cell sorter (BD Biosciences).

**Mammosphere formation assay**

The mammosphere formation assay was performed as described previously^7^. The carcinoma cells were plated into ultralow attachment 24-well plates (Corning) at a density of 5000 cells and grown in serum-free DMEM/Ham’s F12 supplemented with 20 ng/ml bFGF, 20 ng/ml EGF, and B27. Fresh medium of 500 µl was replenished every 3 days during continuous culture for 7 days. The number of spheres greater than 75 µm in diameter was counted under a phase-contrast microscope. For inhibitor treatment, MMTV-PyVT cells were cultured in medium with or without the ST045849 inhibitor for 7 days.

**Aldefluor flow cytometry assay**

The ALDEFLUOR Kit (Stem cell Technologies) was used to measure cell populations with high ALDH activity. Briefly, cancer cells (2 x 10^5^ cells) were suspended in 0.2 ml of ALDEFLUOR assay buffer containing 3 µM BODIPY-aminoacetaldehyde and incubated for 60 min at 37°C. As a negative control, cells were treated with 15 µM diethylaminobenzaldehyde (DEAB), a specific ALDH inhibitor before the reaction and used as nonspecific background staining for the assay to gate the ALDH positive cells. A total of 20,000 viable cells were analyzed using the FACSMelody cell sorter.

**Supplementary figure legends**

**Supplementary Fig. S1** Gene amplification of *HAS2* in aggressive breast cancers. **a** Gene amplification of *HAS2* in clinical breast cancers (data from TCGA Research Network) and Kaplan–Meier curves for overall survival in patients with and without gene amplification. The total number of analyzed samples was 5071 for gene amplification and overall survival. Raw *p*-values were calculated by the log-rank test. **b** Copy number of *HAS2* gene within different cancer types from TCGA PanCancer Atlas dataset (n = 1070). **c** The mRNA expression of HAS2 within different cancer types from TCGA PanCancer Atlas dataset (n = 1070).

**Supplementary Fig. S2** HBP flux regulates *O*-GlcNAcylation and HA production. **a** Western blot analysis for protein *O*-GlcNAcylation after GFAT inhibition. MMTV-PyVT cancer cells were treated with 20 µM GFAT inhibitor (DON) for the indicated times (3–48 h) and cell lysates were subjected to western blot analysis. Band intensities were quantified by densitometric analysis using ImageJ software and standardized with respect to a β-actin internal control. Data represent the mean ± S.D. of three independent experiments. **, *p* < 0.01 versus untreated control cells. **b** HA content in the conditioned medium of MMTV-PyVT cancer cells was measured by a competitive ELISA-like assay. The cells were treated with 20 µM DON or a combination of 20 µM DON and 200 µM D-Glucosamine (GlcN) for 48 h. Data represent the mean ± S.D. of three independent experiments. **, *p* < 0.01.

**Supplementary Fig. S3** Representative ALDH profiles of Has2-deficient Has2^Δ/Δ^ and control Has2^flox/flox^ cells (**a**), GFAT knockdown (shGFAT) and control (shControl) cells (**b**), and OGT inhibitor-treated MMTV-PyVT cells (**c**). ALDH^+^ cells were identified using a flow cytometry-based Aldefluor assay. Baseline fluorescence was established in the presence of the ALDH inhibitor, DEAB (insets). Data represent the mean ± S.D. of four independent experiments. *, *p* < 0.05. **, *p* < 0.01.

**Supplementary Fig. S4** Reduced *O*-GlcNAcylation in GFAT knockdown cells. **a** Western blot analysis for protein *O*-GlcNAcylation in GFAT knockdown (shGFAT) and control (shControl) cells. Band intensities were quantified by densitometric analysis using ImageJ software and standardized with respect to a β-actin internal control. Data represent the mean ± S.D. of five independent experiments. *, *p* < 0.05. **b** Western blot analysis for OGT and OGA expression in shGFAT and shControl cells. Data represent the mean ± S.D. of three independent experiments.

**Supplementary References**

1. Prater, M., Shehata, M., Watson, C. J. & Stingl, J. Enzymatic dissociation, flow cytometric analysis, and culture of normal mouse mammary tissue. *Methods Mol. Biol.* **946**, 395-409 (2013).
2. Koyama, H. et al. Hyperproduction of hyaluronan in neu-induced mammary tumor accelerates angiogenesis through stromal cell recruitment: possible involvement of versican/PG-M. *Am. J. Pathol.* **170**, 1086-1099 (2007).
3. Itano, N. et al. Three isoforms of mammalian hyaluronan synthases have distinct enzymatic properties. *J. Biol. Chem.* **274**, 25085-25092 (1999).
4. Ontong, P., Hatada, Y., Taniguchi, S., Kakizaki, I. & Itano, N. Effect of a cholesterol-rich lipid environment on the enzymatic activity of reconstituted hyaluronan synthase. *Biochem. Biophys. Res. Commun.* **443**, 666-671 (2014).
5. Nakajima, K. et al. Simultaneous determination of nucleotide sugars with ion-pair reversed-phase HPLC. *Glycobiology* **20**, 865-871 (2010).
6. Chanmee, T. et al. Hyaluronan Production Regulates Metabolic and Cancer Stem-like Properties of Breast Cancer Cells via Hexosamine Biosynthetic Pathway-coupled HIF-1 Signaling. *J. Biol. Chem.* **291**, 24105-24120 (2016).
7. Chanmee, T. et al. Excessive hyaluronan production promotes acquisition of cancer stem cell signatures through the coordinated regulation of Twist and the transforming growth factor beta (TGF-beta)-Snail signaling axis. *J. Biol. Chem.* **289**, 26038-26056 (2014).
8. Glück, S. et al. TP53 genomics predict higher clinical and pathologic tumor response in operable early-stage breast cancer treated with docetaxel-capecitabine ± trastuzumab. *Breast Cancer Res. Treat.* **132**, 781-791 (2012).
9. Radvanyi, L. et al. The gene associated with trichorhinophalangeal syndrome in humans is overexpressed in breast cancer. *Proc. Natl. Acad. Sci. USA* **102**, 11005-11010 (2005).
10. Finak, G. et al. Stromal gene expression predicts clinical outcome in breast cancer. *Nat. Med.* **14**, 518-527 (2008).
11. Richardson, A.L. et al. X chromosomal abnormalities in basal-like human breast cancer. *Cancer Cell* **9**, 121-132 (2006).
12. Ma, X.J., Dahiya, S., Richardson, E., Erlander, M. & Sgroi, D.C. Gene expression profiling of the tumor microenvironment during breast cancer progression. *Breast Cancer Res.* **11**, R7 (2009).
13. Curtis, C. et al. The genomic and transcriptomic architecture of 2,000 breast tumours reveals novel subgroups. *Nature* **486**, 346-352 (2012).
14. Turashvili, G. et al. Novel markers for differentiation of lobular and ductal invasive breast carcinomas by laser microdissection and microarray analysis. *BMC Cancer* **7**, 55 (2007).
15. TCGA (The Cancer Genome Atlas). The Cancer Genome Atlas - Invasive Breast Carcinoma Gene Expression Data. <http://tcga-data.nci.nih.gov/tcga/>
16. Zhao, H. et al. Different gene expression patterns in invasive lobular and ductal carcinomas of the breast. *Mol. Biol. Cell* **15**, 2523-2536 (2004).
17. Sørlie, T. et al. Repeated observation of breast tumor subtypes in independent gene expression data sets. *Proc. Natl. Acad. Sci. USA* **100**, 8418-8423 (2003).
18. Sørlie, T. et al. Gene expression patterns of breast carcinomas distinguish tumor subclasses with clinical implications. *Proc. Natl. Acad. Sci. USA* **98**, 10869-10874 (2001).
19. Karnoub, A.E. et al. Mesenchymal stem cells within tumour stroma promote breast cancer metastasis. *Nature* **449**, 557-563 (2007).
