# Supplementary material for: Enhanced hexosamine metabolism drives metabolic and signaling networks involving hyaluronan production and O-GlcNAcylation to exacerbate breast cancer
Source: Cell Death Dis. 2019 Oct 23;10(11):803. doi: 10.1038/s41419-019-2034-y (PMC6811536; doi:10.1038/s41419-019-2034-y)
Supplement: Supplementary file 2 — Supplementary Table S1 [file 41419_2019_2034_MOESM2_ESM.docx]

**Supplementary Table S1. Up-regulation of genes encoding HBP enzymes in clinical breast cancers.**

| **Datasets** |  | **GFAT1/GFAT2** | | **GNPNAT1** | | **PGM3** | | **UAP1** | | **References** |
| --- | --- | --- | --- | --- | --- | --- | --- | --- | --- | --- |
|  |  | Fold | *p*-value | Fold | *p*-value | Fold | *p*-value | Fold | *p*-value |  |
| **Glück Breast** | | | | | | | | | | 8 |
| Invasive breast carcinoma |  | 1.526  (154:4) | 1.12e-4 | 1.687  (154:4) | 0.016 | n.s. |  | n.s. |  |  |
| **Radvanyi Breast** | | | | | | | | | | 9 |
| Invasive ductal breast carcinoma |  | 2.414  (30:6) | 0.044 | 2.933  (31:5) | 1.44e-5 | n.s. |  | n.s. |  |  |
| Invasive lobular breast carcinoma |  | 4.491  (4:5) | 0.046 | 1.937  (7:5) | 0.040 | n.s. |  | n.s. |  |  |
| **Finak Breast** | | | | | | | | | | 10 |
| Invasive breast carcinoma stroma |  | 5.616†  (53:6) | 3.80e-14 | 1.761  (53:6) | 2.55e-6 | n.s. |  | n.s. |  |  |
| **Richardson Breast 2** | | | | | | | | | | 11 |
| Ductal breast carcinoma |  | 2.144  (40:7) | 5.15e-7 | n.s. |  | 2.208  (40:7) | 5.28e-5 | 1.608  (40:7) | 0.012 |  |
| **Ma Breast 4** | | | | | | | | | | 12 |
| Ductal breast carcinoma *in situ* epithelia |  | 2.424  (9:14) | 4.12e-6 | 1.738  (9:14) | 4.29e-4 | 2.514  (9:14) | 2.12e-5 | 1.968  (9:14) | 0.007 |  |
| Ductal breast carcinoma *in situ* stroma |  | 2.360†  (11:14) | 2.13e-5 | n.s. |  | 1.869  (11:14) | 0.002 | n.s. |  |  |
| Invasive ductal breast carcinoma epithelia |  | 1.595  (9:14) | 0.008 | n.s. |  | 1.634  (9:14) | 0.005 | n.s. |  |  |
| Invasive ductal breast carcinoma stroma |  | 2.393†  (9:14) | 4.94e-4 | n.s. |  | 1.900  (9:14) | 0.004 | n.s. |  |  |
| **Curtis Breast** | | | | | | | | | | 13 |
| Ductal breast carcinoma *in situ* |  | 1.506  (10:144) | 0.026 | n.s. |  | 1.600  (10:144) | 3.50e-5 | n.s. |  |  |
| Medullary breast carcinoma |  | 1.514  (32:144) | 5.20e-10 | n.s. |  | n.s. |  | n.s. |  |  |
| Mucinous breast carcinoma |  | 1.829  (46:144) | 3.36e-13 | n.s. |  | n.s. |  | n.s. |  |  |
| Invasive breast carcinoma |  | 1.640  (21:144) | 8.10e-5 | n.s. |  | 1.658  (21:144) | 6.90e-5 | n.s. |  |  |
| Invasive ductal breast carcinoma |  | 1.528  (1,556:144) | 4.48e-40 | n.s. |  | n.s. |  | n.s. |  |  |
| Invasive ductal and invasive lobular breast carcinoma |  | 1.500  (90:144) | 3.08e-14 | n.s. |  | n.s. |  | n.s. |  |  |
| **Turashvili Breast** | | | | | | | | | | 14 |
| Invasive lobular breast carcinoma |  | 2.238†  (5:10) | 0.044 | n.s. |  | n.s. |  | n.s. |  |  |
| **TCGA Breast** | | | | | | | | | | 15 |
| Mucinous breast carcinoma |  | 1.982  (4:61) | 0.013 | n.s. |  | n.s. |  | 1.613  (4:61) | 8.14e-4 |  |
| Mixed lobular and ductal breast carcinoma |  | n.s. |  | 1.617  (7:61) | 2.88e-4 | n.s. |  | n.s. |  |  |
| Male breast carcinoma |  | n.s. |  | 1.917  (3:61) | 0.005 | n.s. |  | n.s. |  |  |
| Invasive breast carcinoma |  | n.s. |  | 1.735  (76:61) | 1.21e-15 | n.s. |  | n.s. |  |  |
| Invasive ductal breast carcinoma |  | n.s. |  | 2.032  (389:61) | 1.70e-29 | n.s. |  | n.s. |  |  |
| Invasive lobular breast carcinoma |  | n.s. |  | 1.565  (36:61) | 2.13e-6 | n.s. |  | n.s. |  |  |
| Invasive ductal and lobular carcinoma |  | n.s. |  | 1.633  (3:61) | 0.006 | n.s. |  | 1.571  (3:61) | 4.76e-4 |  |
| **Zhao Breast** | | | | | | | | | | 16 |
| Lobular breast carcinoma |  | n.s. |  | n.s. |  | 2.414  (18:3) | 3.85e-7 | 1.721  (21:3) | 4.05e-6 |  |
| Invasive ductal breast carcinoma |  | n.s. |  | n.s. |  | 1.667  (38:3) | 4.53e-9 | 1.856  (37:3) | 6.43e-8 |  |
| **Sørlie Breast 2** | | | | | | | | | | 17 |
| Ductal breast carcinoma |  | n.s. |  | n.s. |  | n.s. |  | 1.644  (93:4) | 0.028 |  |
| Lobular breast carcinoma |  | n.s. |  | n.s. |  | n.s. |  | 1.805  (7:4) | 0.046 |  |
| **Sørlie Breast** | | | | | | | | | | 18 |
| Ductal breast carcinoma |  | n.s. |  | n.s. |  | n.s. |  | 1.703  (65:4) | 0.034 |  |

Genes exhibiting ≥ 1.5-log2 fold change of gene expression with *p*-value < 0.05 between breast cancer and normal samples in Oncomine databases (cDNA microarray analysis) are listed. † indicates the data of GFAT2. n.s; not significant. Numbers in parentheses indicate the number of cancer vs. normal samples.
