# Supplementary material for: Enhanced hexosamine metabolism drives metabolic and signaling networks involving hyaluronan production and O-GlcNAcylation to exacerbate breast cancer
Source: Cell Death Dis. 2019 Oct 23;10(11):803. doi: 10.1038/s41419-019-2034-y (PMC6811536; doi:10.1038/s41419-019-2034-y)
Supplement: Supplementary file 3 — Supplementary Table S2 [file 41419_2019_2034_MOESM3_ESM.docx]

**Supplementary Table S2**. **Gene expression of *O*-GlcNAc cycling enzymes in clinical breast cancers.**

| **Datasets** | **OGT** | | | **OGA** | | | **References** |
| --- | --- | --- | --- | --- | --- | --- | --- |
|  | Fold | *p*-value | | Fold | | *p*-value |  |
| **Glück Breast** | | | | | | | 8 |
| Invasive breast carcinoma | 1.656  (154:4) | | 1.37e-4 | | n.s. |  |  |
| **Karnoub Breast** | | | | | | | 19 |
| Invasive ductal breast carcinoma stroma | 1.511  (7:15) | | 0.009 | | n.s. |  |  |
| **Curtis Breast** | | | | | | | 13 |
| Ductal breast carcinoma *in situ* | n.s. | |  | | -2.619  (10:144) | 2.31e-7 |  |
| Benign breast neoplasm | n.s. | |  | | -2.336  (3:144) | 0.02 |  |
| Breast carcinoma | n.s. | |  | | -2.039  (14:144) | 7.04e-6 |  |
| Invasive breast carcinoma | n.s. | |  | | -2.018  (21:144) | 2.23e-13 |  |
| Mucinous breast carcinoma | n.s. | |  | | -1.970  (46:144) | 2.09e-20 |  |
| Medullary breast carcinoma | n.s. | |  | | -1.921  (32:144) | 4.05e-13 |  |
| Invasive ductal breast carcinoma | n.s. | |  | | -1.849  (1,556:144) | 3.69e-88 |  |
| Tubular breast carcinoma | n.s. | |  | | -1.788  (67:144) | 9.31e-36 |  |
| Invasive ductal and invasive lobular breast carcinoma | n.s. | |  | | -1.784  (90:144) | 1.06e-34 |  |
| Breast phyllodes tumor | n.s. | |  | | -1.703  (5:144) | 0.021 |  |
| Invasive lobular breast carcinoma | n.s. | |  | | -1.650  (148:144) | 2.10e-43 |  |
| **Radvanyi Breast** | | | | | | | 9 |
| Invasive ductal breast carcinoma | n.s. | |  | | -1.841  (13:4) | 0.050 |  |
| **Richardson Breast 2** | | | | | | | 11 |
| Ductal breast carcinoma | n.s. | |  | | -1.773  (40:7) | 3.17e-7 |  |
| **TCGA Breast** | | | | | | | 15 |
| Invasive breast carcinoma | n.s. | |  | | -1.766  (76:61) | 1.05e-10 |  |
| Invasive ductal breast carcinoma | n.s. | |  | | -1.684  (389:61) | 4.41e-13 |  |

Genes exhibiting ≥ 1.5-log2 fold change of gene expression with *p*-value < 0.05 between breast cancer and normal samples in Oncomine databases (cDNA microarray analysis) are listed. n.s; not significant. Numbers in parentheses indicate the number of cancer vs. normal samples.
