# Supplementary figures and images for: Enhanced hexosamine metabolism drives metabolic and signaling networks involving hyaluronan production and O-GlcNAcylation to exacerbate breast cancer
Source: Cell Death Dis. 2019 Oct 23;10(11):803. doi: 10.1038/s41419-019-2034-y (PMC6811536; doi:10.1038/s41419-019-2034-y)

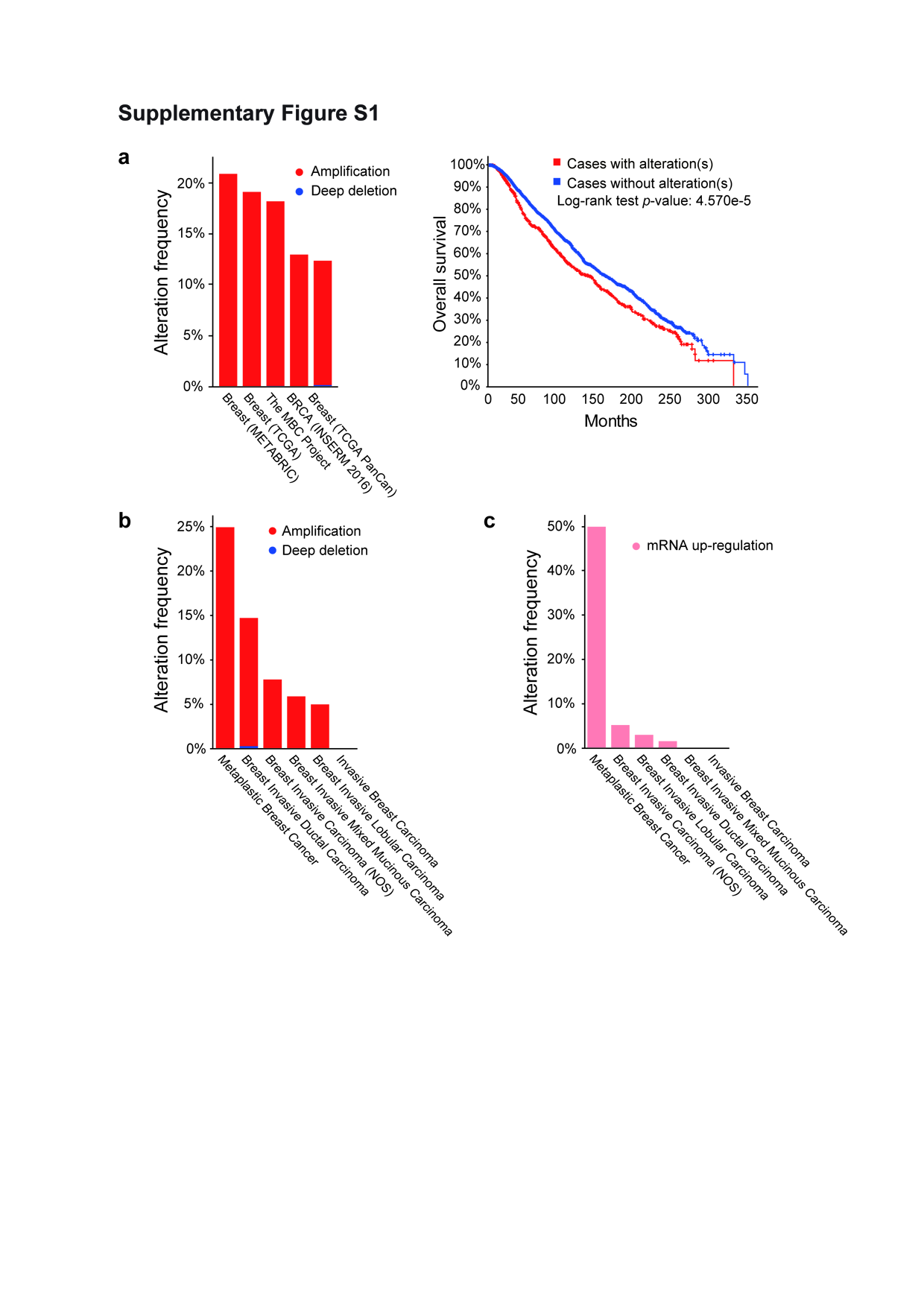

Supplement: Supplementary file 4 — Supplementary Figure S1 [file 41419_2019_2034_MOESM4_ESM.tif]

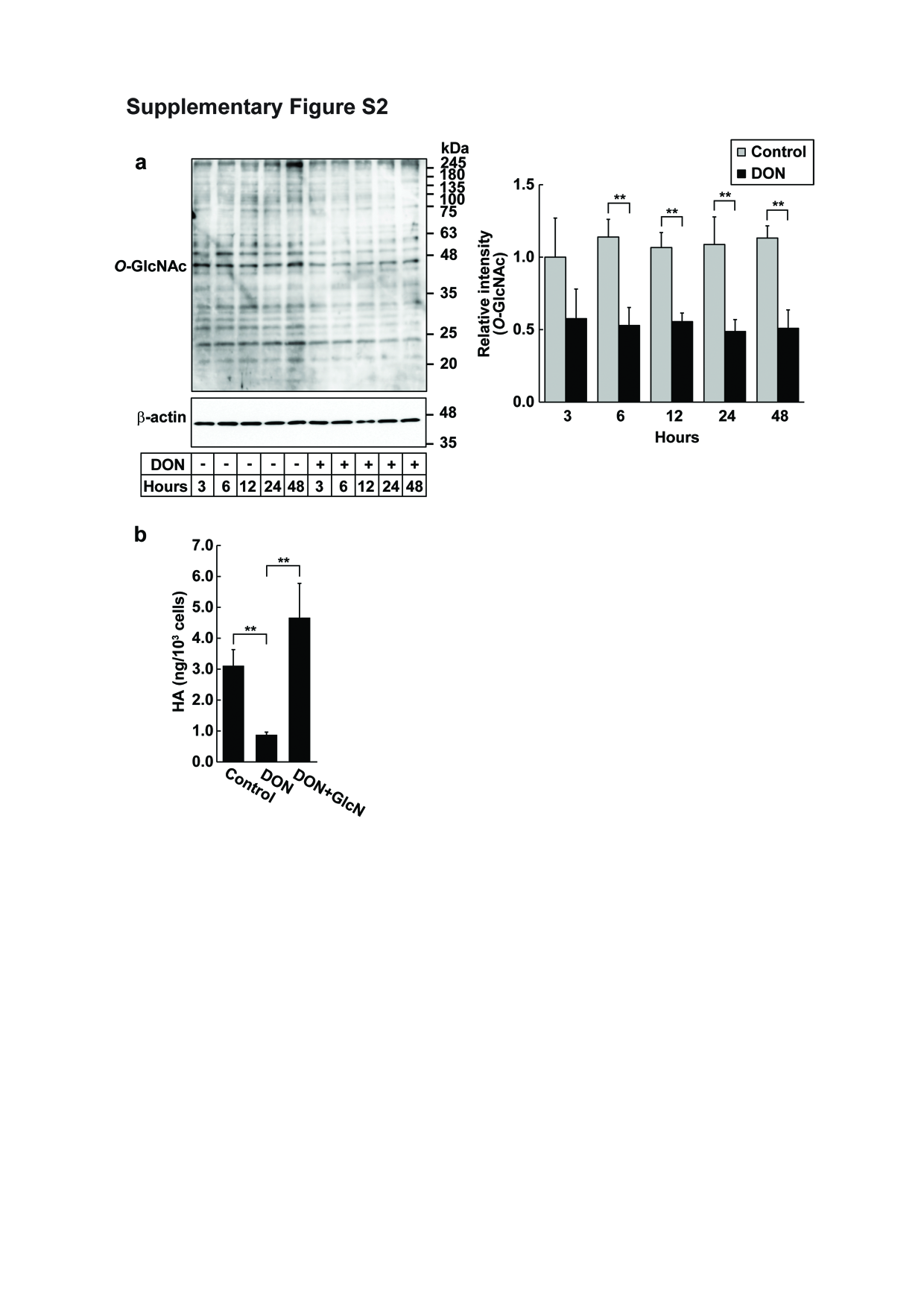

Supplement: Supplementary file 5 — Supplementary Figure S2 [file 41419_2019_2034_MOESM5_ESM.tif]

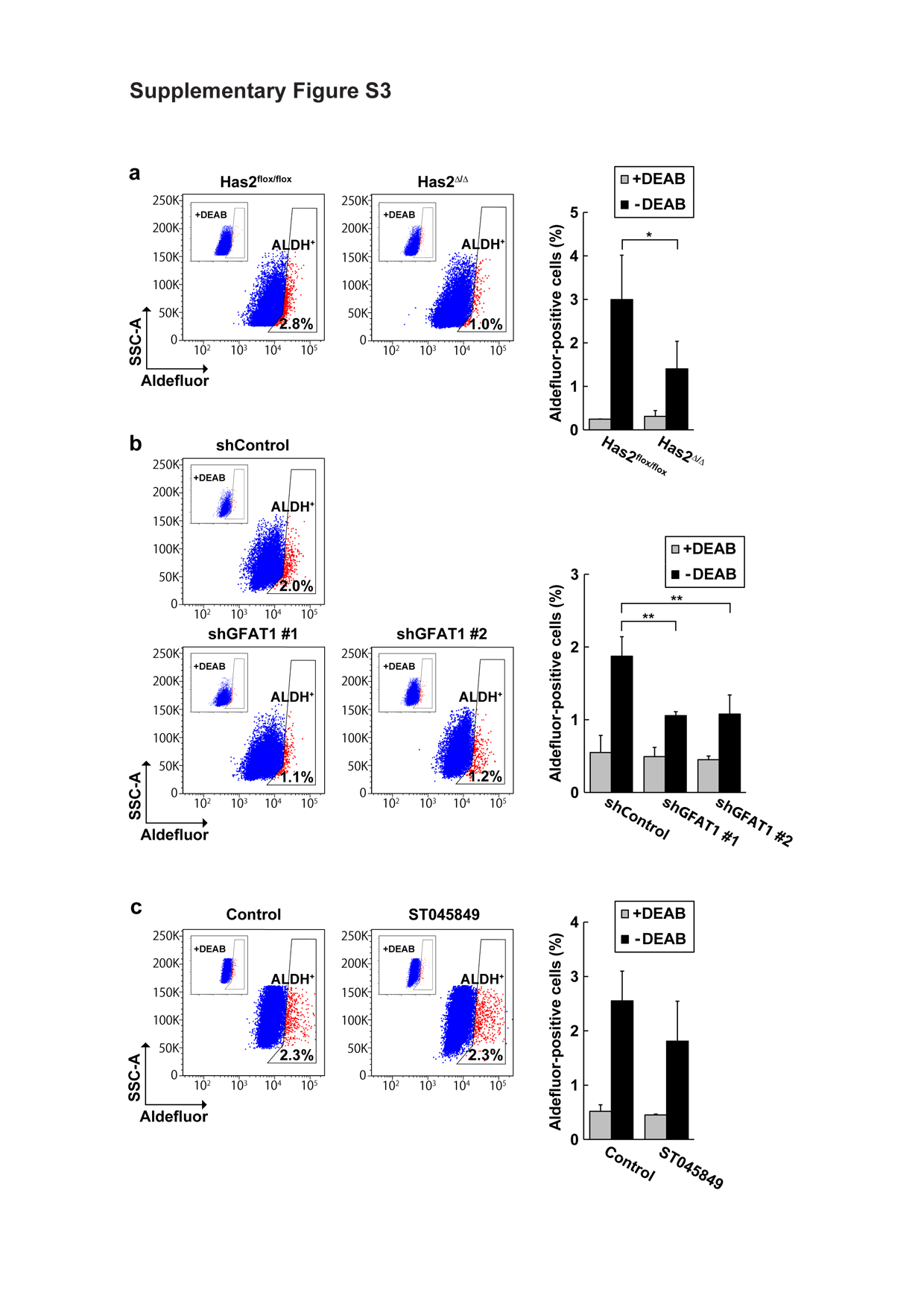

Supplement: Supplementary file 6 — Supplementary Figure S3 [file 41419_2019_2034_MOESM6_ESM.tif]

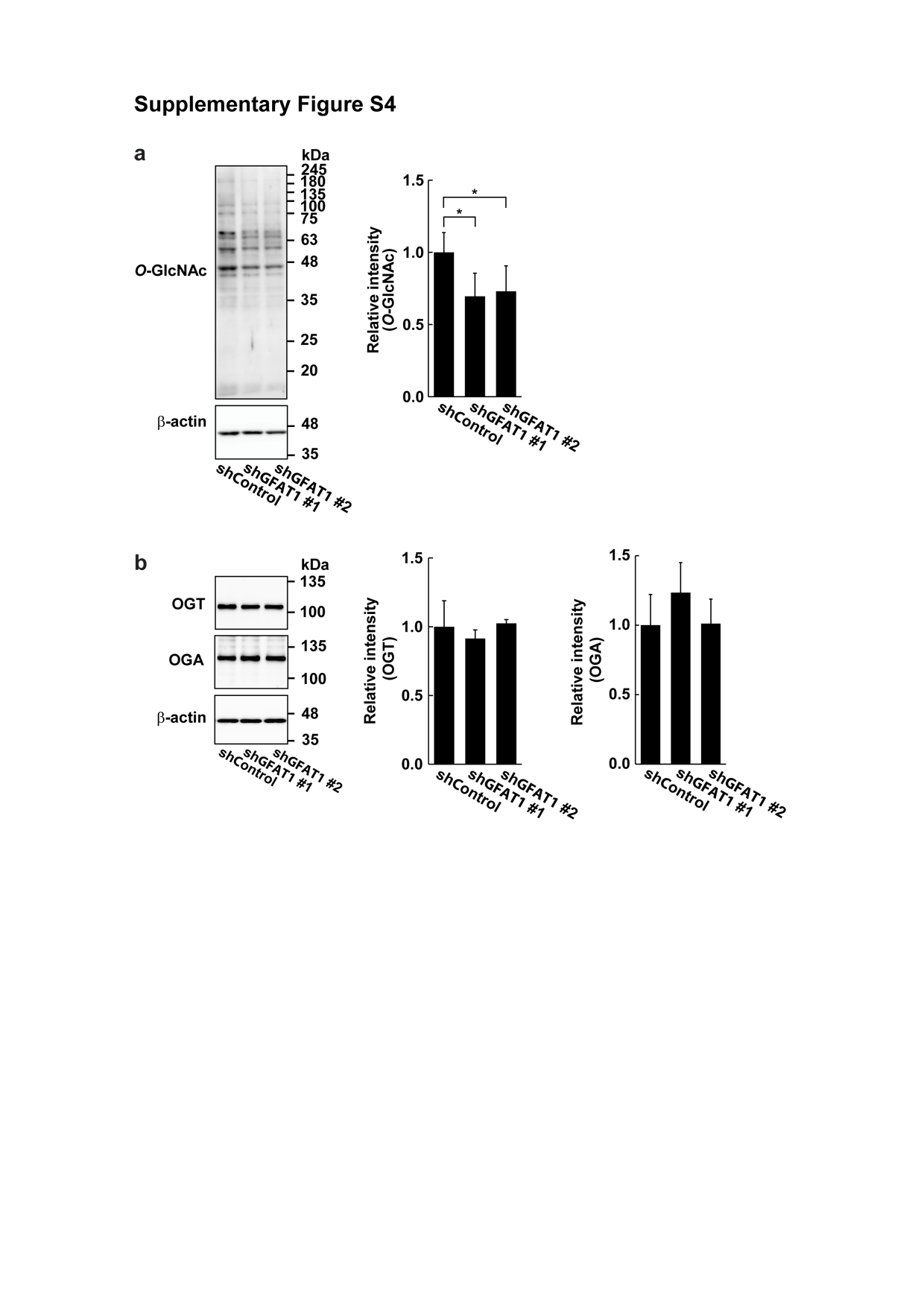

Supplement: Supplementary file 7 — Supplementary Figure S4 [file 41419_2019_2034_MOESM7_ESM.tif]
