# Supplementary material for: Enhanced hexosamine metabolism drives metabolic and signaling networks involving hyaluronan production and O-GlcNAcylation to exacerbate breast cancer
Source: Cell Death Dis. 2019 Oct 23;10(11):803. doi: 10.1038/s41419-019-2034-y (PMC6811536; doi:10.1038/s41419-019-2034-y)
Supplement: Supplementary file 8 — Detailed Attribution of Authorship [file 41419_2019_2034_MOESM8_ESM.pdf]

DECLARATION OF CONTRIBUTIONS TO ARTICLE

ADMC

Manuscript Number: CDDIS-19-0317

Journal Name: *Cell Death & Differentiation* (the 'Journal')

Proposed Title of the Contribution:

Enhanced hexosamine metabolism drives metabolic and signaling networks involving hyaluronan production and O-GlcNAcylation to exacerbate breast cancer. (the 'Contribution)

Author(s):

Chatchadawalai Chokchaitaweesuk, Takashi Kobayashi, Tomomi Izumikawa, Naoki Itano (the 'Authors')

For all *CDD* articles, each person named as an author in the published version must be able to show he or she has contributed substantially to the article.

Authorship credit should be based on 1) substantial contributions to conception and design, acquisition of data, or analysis and interpretation of data; 2) drafting the article or revising it critically for important intellectual content; and 3) final approval of the version to be published. Authors should meet conditions 1, 2 and 3.

Any person who cannot be shown to have made a substantial contribution to the article cannot be listed as an author in the final version. The name of any person who is deemed to have made a minor contribution can, however, appear in the Acknowledgments section of the article.

Please complete the table below to indicate the contributions of all named authors to the manuscript.

Author Full Name:	Specification of Contribution to the Manuscript:
CC	Acquisition and analysis of data, Construct of figures
TK	Acquisition and analysis of data, Assist the preparation of manuscript
TI	Acquisition and analysis of data
NI	Design of the study, Acquisition and analysis of data, Writing the manuscript

Please complete the table below to indicate the contributions of all named authors to the figures.

Figure 1:

TK analyzed the data and prepared panels, NI assembled the figure.

Figure 2:

CC generated the data and prepared panel d, TK generated the data and prepared panels a-c and e, NI generated the data of panel f, NI assembled the figure.

Figure 3:

CC generated the data and prepared panels a-d, TK and TI generated the data and prepared panel e, NI assembled the figure.

Figure 4:

CC generated the data and prepared panels, NI assembled the figure.

Figure 5:

CC generated the data and prepared panels a, c, e, f ,TK generated the data and prepared panel b, d, NI assembled the figure.

Figure 6:

CC and TI generated the data and prepared panels, NI assembled the figure.

Signed for and on behalf of the Author(s):	Print Name:	Date:
Naoki Itano	Naoki Itano	September 25, 2019
