# Supplementary material for: Enhanced hexosamine metabolism drives metabolic and signaling networks involving hyaluronan production and O-GlcNAcylation to exacerbate breast cancer
Source: Cell Death Dis. 2019 Oct 23;10(11):803. doi: 10.1038/s41419-019-2034-y (PMC6811536; doi:10.1038/s41419-019-2034-y)
Supplement: Supplementary file 9 — Additional Detailed Attribution of Authorship [file 41419_2019_2034_MOESM9_ESM.docx]

**Declaration of contributions to article (*continued*)**

**Enhanced hexosamine metabolism drives metabolic and signaling networks involving hyaluronan production and O-GlcNAcylation to exacerbate breast cancer.**

Chatchadawalai Chokchaitaweesuk, Takashi Kobayashi, Tomomi Izumikawa & Naoki Itano

**Figure 7:**

CC generated the data and prepared panels a and b, TK and TI generated the data and prepared panel c, NI assembled the figure.

**Table 1:**

TK generated the data, NI assembled the table.

**Supplementary Figure S1:**

NI analyzed the data and prepared panels, NI assembled the figure.

**Supplementary Figure S2:**

CC generated the data and prepared panels, NI assembled the figure.

**Supplementary Figure S3:**

TK generated the data and prepared panels, NI assembled the figure.

**Supplementary Figure S4:**

TK generated the data and prepared panel a, CC generated the data and prepared panel b, NI assembled the figure.

**Supplementary Table S1:**

NI generated the data and assembled the table.

**Supplementary Table 2:**

NI generated the data and assembled the table.
